# Discovery of novel antituberculosis agents among 3-phenyl-5-(1-phenyl-1H-[1,2,3]triazol-4-yl)-[1,2,4]oxadiazole derivatives targeting aminoacyl-tRNA synthetases

**DOI:** 10.1038/s41598-021-86562-y

**Published:** 2021-03-30

**Authors:** Mariia Yu. Rybak, Anatoliy O. Balanda, Anna P. Yatsyshyna, Igor. M. Kotey, Sergiy A. Starosyla, Volodymyr G. Bdzhola, Lubov L. Lukash, Sergiy M. Yarmoluk, Michael A. Tukalo, Galyna P. Volynets

**Affiliations:** 1 Department of Protein Synthesis Enzymology, Institute of Molecular Biology and Genetics of the NAS of Ukraine, Kyiv, Ukraine; 2 Department of Medicinal Chemistry, Institute of Molecular Biology and Genetics of the NAS of Ukraine, Kyiv, Ukraine; 3 Department of Human Genetics, Institute of Molecular Biology and Genetics of the NAS of Ukraine, Kyiv, Ukraine

**Keywords:** Chemical biology, Microbiology, Molecular biology, Microbiology techniques, Cell culture, Biochemical assays, Biochemistry, Enzymes, Drug discovery, Medicinal chemistry

## Abstract

Antibiotic resistance is a major problem of tuberculosis treatment. This provides the stimulus for the search of novel molecular targets and approaches to reduce or forestall resistance emergence in *Mycobacterium tuberculosis*. Earlier, we discovered a novel small-molecular inhibitor among 3-phenyl-5-(1-phenyl-1H-[1,2,3]triazol-4-yl)-[1,2,4]oxadiazoles targeting simultaneously two enzymes—mycobacterial leucyl-tRNA synthetase (LeuRS) and methionyl-tRNA synthetase (MetRS), which are promising molecular targets for antibiotic development. Unfortunately, the identified inhibitor does not reveal antibacterial activity toward *M. tuberculosis*. This study aims to develop novel aminoacyl-tRNA synthetase inhibitors among this chemical class with antibacterial activity toward resistant strains of *M. tuberculosis*. We performed molecular docking of the library of 3-phenyl-5-(1-phenyl-1H-[1,2,3]triazol-4-yl)-[1,2,4]oxadiazole derivatives and selected 41 compounds for investigation of their inhibitory activity toward MetRS and LeuRS in aminoacylation assay and antibacterial activity toward *M. tuberculosis* strains using microdilution assay. In vitro screening resulted in 10 compounds active against MetRS and 3 compounds active against LeuRS. Structure-related relationships (SAR) were established. The antibacterial screening revealed 4 compounds active toward *M. tuberculosis* mono-resistant strains in the range of concentrations 2–20 mg/L. Among these compounds, only one compound **27** has significant enzyme inhibitory activity toward mycobacterial MetRS (IC_50_ = 148.5 µM). The MIC for this compound toward *M. tuberculosis* H37Rv strain is 12.5 µM. This compound is not cytotoxic to human HEK293 and HepG2 cell lines. Therefore, 3-phenyl-5-(1-phenyl-1H-[1,2,3]triazol-4-yl)-[1,2,4]oxadiazole derivatives can be used for further chemical optimization and biological research to find non-toxic antituberculosis agents with a novel mechanism of action.

## Introduction

Tuberculosis is one of the most dangerous infectious diseases and a serious social problem. The main problem of tuberculosis treatment is multidrug resistance to existing antibiotics. Therefore, the search for novel molecular targets and the development of novel strategies to overcome *M. tuberculosis* resistance became a challenging task for modern science.

Nowadays, aminoacyl-tRNA synthetases (ARSases) represent promising molecular targets for antibiotic development^1–4^. These enzymes catalyze the covalent attachment of amino acid residues to cognate tRNAs, playing a key role in the first stage of protein biosynthesis. Aminoacyl-tRNA synthetases have a highly conservative structure among different pathogens, which increases the chances of antibiotic development with a broad spectrum of action. At the same time, aminoacyl-tRNA synthetases possess some structural divergence between prokaryotes and eukaryotes which can be sufficient for the development of inhibitors with higher selectivity toward pathogen enzymes. Besides, several pro- and eukaryotic aminoacyl-tRNA synthetases also have differences in aminoacyl-adenylate binding sites in comparison with human mitochondrial ARSases, this may significantly reduce the toxicity of targeting aminoacyl-adenylate binding site inhibitors during antibiotic therapy.

In particular, *M. tuberculosis* leucyl-tRNA synthetase (LeuRS) has significant differences in the amino acid sequences of the active sites compared to the human enzyme^5^. Recently, an inhibitor of mycobacterial LeuRS was found among benzoxaborole derivatives – GSK656, which is highly selective toward mycobacterial LeuRS (IC_50_ = 0.2 µM) compared to human cytoplasmic (IC_50_ > 300 µM) and mitochondrial LeuRS (IC_50_ > 300 µM). Besides, GSK656 is effective in an animal model of tuberculosis and is recommended for clinical trials, indicating that *M. tuberculosis* LeuRS is a validated molecular target for the development of anti-tuberculosis drugs^6^.

*M. tuberculosis* methionyl-tRNA synthetase (MetRS) differs in amino acid residues of aminoacyl-adenylate binding sites by 44% and 22% from human cytosolic and mitochondrial MetRS, correspondingly. It should be noted that inhibitor of *Staphylococcus aureus* MetRS – REP8839, has a 1000-fold higher affinity for the bacterial enzyme than for human mitochondrial MetRS^7^, while the amino acid difference in the active site between *S. aureus* and human mitochondrial MetRS is the same as in the case of mycobacterial and human mitochondrial MetRS. With the fact that considerable selectivity between *S. aureus* and human mitochondrial enzymes was achieved, there are opportunities to develop inhibitors with higher selectivity toward *M.* *tuberculosis* MetRS than for both human homologs. Besides, recently several crystal structures of *M. tuberculosis* MetRS were reported^8,9^, which makes this enzyme promising for the receptor-oriented rational design of inhibitors with antituberculosis activity. Unfortunately, the frequency of resistance occurrence to aaRSs inhibitors is very high (10^–7^–10^–8^) due to point mutations in the gene encoding enzyme which consequently affects ligand-binding interactions.

Several experimental studies suggest that MetRS is a validated molecular target for antibiotic design^10–20^. However, to date, there are only several published reports of small molecular inhibitors for mycobacterial MetRS among some adenylate analogs^16^ and previously published by us N-benzylidene-N′-thiazol-2-yl-hydrazine^21^ and 3-Phenyl-5-(1-phenyl-1H-[1,2,3]triazol-4-yl)-[1,2,4]oxadiazole^22^ derivatives. Moreover, these derivatives represent first-published dual-target inhibitors of mycobacterial ARSes, inhibiting also LeuRS. However, ARSases inhibitors among N-benzylidene-N′-thiazol-2-yl-hydrazines have poor solubility and reported inhibitor 3-(3-chloro-4-methoxy-phenyl)-5-[3-(4-fuoro-phenyl)-[1,2,4]oxadiazol5-yl]-3H-[1,2,3]triazol-4-ylamine does not reveal antibacterial activity. Therefore, the search for novel *M. tuberculosis* MetRS inhibitors with antibacterial activity is very important.

Recently, as an approach to overcome resistance, we have proposed an idea for the development of dual-targeted inhibitors toward *M. tuberculosis* LeuRS and MetRS since mutations are required in all targets to confer resistance to the drug^21^. We identified the first dual-targeted inhibitors toward mycobacterial LeuRS and MetRS among N-benzylidene-N’-thiazol-2-yl-hydrazine^21^, which previously were reported as inhibitors of LeuRS^5^. Moreover, using the method of ligand-based pharmacophore modeling we have found 5 novel hit compounds possessing inhibitory activity simultaneously toward *M. tuberculosis* LeuRS and MetRS^22^. The most active compound—3-(3-chloro-4-methoxy-phenyl)-5-[3-(4-fluoro-phenyl)-[1,2,4]oxadiazol-5-yl]-3H-[1,2,3]triazol-4-ylamine inhibits activity of mycobacterial LeuRS and MetRS with IC_50_ values of 13 µM and 13.8 µM, respectively. Unfortunately, this compound does not have antibacterial activity toward *M.* *tuberculosis*. Therefore, the search for novel LeuRS and MetRS inhibitors among this chemical class with antituberculosis activity is of great interest.

## Results

To find novel MetRS and LeuRS inhibitors with the antituberculosis activity we performed molecular docking of the pre-selected compound library of 3-phenyl-5-(1-phenyl-1H-[1,2,3]triazol-4-yl)-[1,2,4]oxadiazole derivatives into active sites of investigated enzymes. According to molecular docking results and visual analysis of the best-scored complexes, we selected 41 compounds for investigation of their inhibitory activity toward recombinant mycobacterial LeuRS and MetRS. The results of testing are presented in Table 1.

**Table 1:** Structures and in vitro activity for 3-Phenyl-5-(1-phenyl-1H-[1,2,3]triazol-4-yl)-[1,2,4]oxadiazole derivatives toward *M. tuberculosis* leucyl-tRNA synthetase (LeuRS) and methionyl-tRNA synthetase (MetRS).


№	R^1^	R^2^	R^3^	R^4^	R^5^	R^6^	R^7^	R^8^	LeuRS inhibition activity, %	MetRS inhibition activity, %
1	H	H	H	F	H	H	H	NH_2_	− 16 ± 3 (2)	− 35 ± 5 (2)
2	H	H	H	H	Cl	CF_3_	H	NH_2_	32 ± 19 (3)	71 ± 29 (2)
3	H	H	F	H	Cl	CF_3_	H	NH_2_	− 22 ± 16 (3)	36 ± 23 (2)
4	H	H	F	H	Cl	F	H	CH_3_	11 ± 8 (3)	− 32 ± 15 (2)
5	H	H	F	H	H	Br	H	CH_3_	17 ± 10 (2)	− 50 ± 9 (2)
6	H	H	F	F	H	H	F	CH_3_	− 26 ± 11 (2)	− 46 ± 3 (2)
7	H	H	Cl	H	F	H	H	CH_3_	71 ± 5 (2)	− 36 ± 25 (2)
8	H	H	CH_3_	H	H	Br	H	CH_3_	18 ± 4 (2)	− 35 ± 1 (2)
9	F	H	H	H	H	H	H	NH_2_	1 ± 8 (2)	30 ± 2 (2)
10	F	H	H	H	H	Cl	H	NH_2_	− 2 ± 2 (3)	23 ± 25 (3)
11	F	H	H	H	H	Br	H	NH_2_	− 12 ± 44 (2)	− 7 ± 13 (2)
12	F	H	H	H	H	F	H	NH_2_	3 ± 7 (2)	52 ± 6 (2)
13	F	H	H	H	H	OCH_3_	H	NH_2_	79 ± 7 (2)	36 ± 5 (2)
14	F	H	H	H	H	CH_3_CH_2_	H	NH_2_	31 ± 7 (2)	41 ± 10 (3)
15	F	H	H	H	F	H	H	NH_2_	71 ± 5 (2)	− 36 ± 25 (2)
16	F	H	H	H	Cl	H	H	NH_2_	− 22 ± 5 (2)	47 ± 19 (2)
17	F	H	H	H	Cl	OCH_3_	H	NH_2_	51 ± 28 (2)	77 ± 3 (2)
18	F	H	H	H	CH_3_	H	H	NH_2_	13 ± 11 (2)	26 ± 20 (2)
19	F	H	H	H	CF_3_	H	H	NH_2_	− 0.7 ± 5 (2)	53 ± 0.7 (2)
20	F	H	H	H	OCH_3_	H	H	NH_2_	29 ± 25 (2)	37 ± 17 (3)
21	F	H	H	H	OCH_3_	OCH_3_	H	NH_2_	− 31 ± 15 (3)	− 32 ± 9 (2)
22	F	H	H	F	H	F	H	NH_2_	− 8 ± 11 (2)	59 ± 9 (2)
23	F	H	H	F	H	H	H	NH_2_	5 ± 7 (2)	7 ± 13 (2)
24	F	H	H	CH_3_	Cl	H	H	NH_2_	− 18 ± 10 (2)	7 ± 21 (2)
25	F	H	H	CH_3_	H	H	Cl	NH_2_	12 ± 12 (2)	25 ± 12 (2)
26	F	H	H	OCH_3_	H	H	H	NH_2_	− 29 ± 4 (2)	− 2 ± 3 (2)
27	F	H	H	OCH_3_	H	H	Cl	NH_2_	14 ± 12 (2)	57 ± 11 (3)
28	F	H	H	OCH_3_	H	H	CH_3_	NH_2_	− 15 ± 10 (2)	37 ± 3 (2)
29	F	H	H	OCH_3_	H	H	OCH_3_	NH_2_	− 22 ± 9 (2)	14 ± 14 (2)
30	F	H	H	OCH_2_CH_3_	H	H	H	NH_2_	− 3 ± 4 (2)	19 ± 0.9 (2)
31	Cl	H	H	F	H	Br	H	NH_2_	15 ± 11 (2)	70 ± 19 (2)
32	Br	H	H	H	H	F	H	NH_2_	− 0.7 ± 15 (2)	46 ± 32 (2)
33	Br	H	H	H	F	H	H	NH_2_	− 18 ± 43 (2)	55 ± 4 (2)
34	Br	H	H	F	H	H	H	NH_2_	17 ± 5 (2)	70 ± 14 (2)
35	Br	H	H	OCH_3_	H	H	H	NH_2_	− 0.23 ± 3 (2)	61 ± 47 (3)
36	Br	H	H	OCH_2_CH_3_	H	H	H	NH_2_	− 13 ± 4 (2)	25 ± 5 (2)
37	OCH_3_	H	H	OCH_3_	H	H	Cl	NH_2_	− 22 ± 0.7 (2)	− 68 ± 16 (2)
38	OCH_3_	OCH_3_	H	H	H	Cl	H	CH_3_	− 34 ± 32 (2)	25 ± 18 (2)
39	OCH_2_CH_3_	H	H	F	H	H	H	NH_2_	− 2 ± 0.2 (2)	35 ± 6 (2)
40	OCH_2_CH_3_	H	H	Cl	H	H	H	NH_2_	36 ± 16 (3)	22 ± 14 (2)
41	OCH_2_CH_3_	H	H	H	Cl	F	H	NH_2_	− 47 ± 28 (2)	59 ± 22 (2)

Recombinant *M. tuberculosis* LeuRS and MetRS were expressed in *Escherichia coli* and purified according to previously described methods: for LeuRS and MetRS^22^, respectively. 3-Phenyl-5-(1-phenyl-1H-[1,2,3]triazol-4-yl)-[1,2,4]oxadiazole derivatives were provided by OTAVA Ltd (Kyiv, Ukraine). The activity of compounds toward mycobacterial LeuRS and MetRS was determined in aminoacylation assay using BIOMOL GREEN reagent (Enzo Life Sciences)^21^ . According to screening data, 10 compounds were decreasing the activity of mycobacterial MetRS and 3 compounds targeting mycobacterial LeuRS with an inhibition cut-off taken as 50%. For compounds that inhibited both synthetases IC_50_ values were determined (Table 2). Noticeably, compounds **2**, **13**, **27** had higher values than previously reported for compound **17**.

**Table 2:** IC_50_ values of dual-target compounds against *M. tuberculosis* LeuRS and MetRS.

№	IC_50_ (µM)	
	LeuRS	MetRS
2	76.5 ± 66	115 ± 3
13	263.5 ± 0.7	122 ± 26
17*	13	13.8
27	ND	148.5 ± 33.2

The shown data represent mean values ± s.d. (n = 3).

*Data of IC_50_ for compound **17** from the previous article^22^.

*ND *not determined.

All these compounds were tested for antibacterial activity toward *M. tuberculosis* H37Rv, a rifampin-resistant strain H37Rv (*rpoB*^S450L^) (ATCC #35,838), an isoniazid-resistant H37Rv (*katG*^del^) (MS015), a moxifloxacin-resistant strain H37Rv (*gyrA*^D94K^) (MOX3), *Mycobacterium avium* (ATCC 700,891 MAC 101) and *Mycobacterium abscessus* (ATCC 19,977) in microdilution assays. Compounds were screened in high-throughput way at 20 mg/L, 2 mg/L, and 0.2 mg/L concentrations and activity determined as > 80% reduction in growth. Growth inhibition values for all tested compounds are listed in Table 3.

**Table 3:** Growth inhibition cutoffs of compounds against *M. tuberculosis* strain panel.

№	H37Rv	H37Rv (*rpoB*^S450L^)	H37Rv (*katG*^del^)	H37Rv (*gyrA*^D94K^)	*M. abscessus* 19,977	*M. avium* 700,891 (MAC 101)
1	> 20	> 20	> 20	> 20	> 20	> 20
2	> 20	> 20	> 20	> 20	> 20	> 20
3	> 20	> 20	> 20	> 20	> 20	> 20
4	> 20	> 20	> 20	> 20	> 20	> 20
5	> 20	2–20	> 20	2–20	> 20	> 20
6	> 20	> 20	> 20	> 20	> 20	> 20
7	> 20	> 20	> 20	> 20	> 20	> 20
8	> 20	> 20	> 20	> 20	> 20	> 20
9	> 20	> 20	> 20	> 20	> 20	> 20
10	> 20	> 20	> 20	> 20	> 20	> 20
11	> 20	> 20	> 20	> 20	> 20	> 20
12	> 20	> 20	> 20	> 20	> 20	> 20
13	> 20	> 20	> 20	> 20	> 20	> 20
14	> 20	> 20	> 20	> 20	> 20	> 20
15	> 20	> 20	> 20	> 20	> 20	> 20
16	> 20	> 20	> 20	> 20	> 20	> 20
17	> 20	> 20	> 20	> 20	> 20	> 20
18	> 20	> 20	> 20	> 20	> 20	> 20
19	> 20	> 20	> 20	> 20	> 20	> 20
20	> 20	2–20	> 20	2–20	> 20	> 20
21	> 20	> 20	> 20	> 20	> 20	> 20
22	> 20	> 20	> 20	> 20	> 20	> 20
23	> 20	> 20	> 20	> 20	> 20	> 20
24	> 20	> 20	> 20	> 20	> 20	> 20
25	> 20	> 20	> 20	> 20	> 20	> 20
26	> 20	> 20	> 20	> 20	> 20	> 20
27	> 20	2–20	> 20	> 20	> 20	> 20
28	> 20	> 20	> 20	> 20	> 20	> 20
29	> 20	> 20	> 20	> 20	> 20	> 20
30	> 20	> 20	> 20	> 20	> 20	> 20
31	> 20	> 20	> 20	> 20	> 20	> 20
32	> 20	> 20	> 20	> 20	> 20	> 20
33	> 20	> 20	> 20	> 20	> 20	> 20
34	> 20	> 20	> 20	> 20	> 20	> 20
35	> 20	> 20	> 20	> 20	> 20	> 20
36	> 20	> 20	> 20	> 20	> 20	> 20
37	> 20	> 20	> 20	> 20	> 20	> 20
38	> 20	> 20	> 20	> 20	> 20	> 20
39	> 20	> 20	> 20	> 20	> 20	> 20
40	> 20	> 20	> 20	2–20	> 20	> 20
41	> 20	> 20	> 20	> 20	> 20	> 20

According to the data from Tables 3, 4 compounds have activity between 2 and 20 mg/L toward several mono-resistant strains. Among these compounds, only one compound **27** (with growth inhibition cutoff 5.17–51.7 µM) has significant enzyme inhibitory activity toward mycobacterial MetRS. Compounds **40** (5.22–52.2 µM cutoff) and **20** (5.7–57 µM) have slight dual-target inhibitory activity toward both *M. tuberculosis* MetRS and LeuRS.

**Table 4:** MIC values for compound **27** against *M. tuberculosis* H37Rv strain in different conditions.

Medium	1-week MIC, µM	2-week MIC, µM
7H9/glucose/casitone/Tx	12.5	25
7H9/glucose/BSA/Tx	> 50	> 50
7H9/DPPC/casitone/Tx	25	25
7H9/DPPC/cholesterol/BSA/Tx	> 50	> 50

The compound **27** was taken for MIC determination toward H37Rv strain in Middlebrook 7H9-based media containing either glucose or cholesterol/dipalmitoylphosphatidylcholine (DPPC) as a carbon source in the presence of bovine serum albumin (BSA) or casitone and supplemented with Tyloxapol. MIC1 and MIC2 values were determined after incubation for 7 and 14 days, correspondingly. As it can be seen from the Table 4, compound **27** inhibits the growth of *M. tuberculosis* H37Rv in the medium containing glucose as a carbon source with 1-week MIC value of 12.5 µM and 2-week MIC value of 25 µM and in the medium containing DPPC as a carbon source with 1-week and 2-week MIC values of 25 µM. In the media containing BSA, the compounds did not inhibit the growth of *M. tuberculosis* H37Rv. It may be explained by the potency them to bind with hydrophobic pockets of free BSA which leads to the decrease of the efficient concentrations of compounds capable to bind with aminoacyl-tRNA synthetases. Similar insights were shown with molecular simulations of human serum albumin (HSA) and three clinically promising squalenoylated drugs (gemcitabine-squalene, adenine-squalene, and doxorubicin-squalene). Data suggest that these drugs may accumulate by HSA and inside low-density lipoproteins^23^. DPPC forms spontaneously vesicle-like structures^24^, thus it may act as the scavenger for different hydrophobic and amphiphilic molecules, similar to lipoproteins^23^.

We have tested compound **27** for cytotoxicity toward human cell lines HEK293 (human embryonic kidney 293) and HepG2 (human hepatocellular carcinoma cell line) using standard MTT assay. According to the results of *in cellulo* testing, this compound did not affect cell viability in the range of concentrations from 3.125 µM to 50 µM, suggesting that CC_50_, concentration to cause 50% cytotoxicity, for HEK293 and HepG2 > 50 µM.

## Discussion

In this study, we have investigated 41 derivatives of 3-Phenyl-5-(1-phenyl-1H-[1,2,3]triazol-4-yl)-[1,2,4]oxadiazole for inhibitory activity toward *M. tuberculosis* MetRS and LeuRS and established several structure–activity relationships (SAR). It has been observed that the nature of R^1^ substituent significantly influences the compound’s inhibitory activity toward *M. tuberculosis* LeuRS and MetRS. We found that compounds with R^1^ = bromine atom are more active than those with an ethoxy group, fluorine, or hydrogen in this position. It can be seen from the comparison of activity for compounds **34**, **39**, **23,** and **1** and compound pairs such as **36**, **30,** and **35**, **26**. From the obtained results, the order of potency for the substituent R^1^ could be proposed as following: Br ≥ CH_3_CH_2_COOH > F > H. Fluorine and bromine atoms have almost equal activity toward mycobacterial LeuRS and MetRS for compound pair **12** and **32**. In the case of compound pair **15** and **33**, the bromine atom is more favored than the fluorine atom for inhibitory activity toward *M. tuberculosis* MetRS but in a case of mycobacterial LeuRS, a fluorine atom is more profitable.

It was revealed that the R^3^ significantly affects the compound’s inhibitory activity toward mycobacterial MetRS and LeuRS. The introduction of fluorine atom instead of Hydrogen in this position leads to a significant decrease of inhibitory activity toward LeuRS and MetRS. To see this effect one can refer to compound pair **2** and **3**.

We have investigated that the substituent R^4^ has also influenced the inhibitory activity toward mycobacterial LeuRS and MetRS. For a pair of compounds with R^1^ = F and R^7^ = Cl, R^4^ = methoxy group (**27**) is more favored than the methyl group (**25**). For a pair of compounds with R^1^ = F and R^7^ = H, R^4^ = ethoxy group (**30**) is more profitable than the methoxy group (**26**) for both enzymes. In a series of compounds with R^1^ = Br (**34**–**36**), the order of inhibitory activity for both enzymes is the following: F < OCH_3_ < OCH_2_CH_3_. For a pair of compounds with R^1^ = OCH_2_CH_3_ (**39–40**), the Cl atom at the position R^4^ plays an important role for dual-targeted inhibition activity toward LeuRS and MetRS and consequently for antibacterial activity toward *M. tuberculosis* resistant strains.

It was found that R^5^ also impacts the compound’s inhibitory activity toward mycobacterial MetRS and LeuRS. The presence of a methoxy group (compound **20**) is more favored than the methyl group (compound **18**) in this position for inhibitory activity toward both enzymes. The introduction of substituents CF_3_ (**19**) or Cl (**16**) in this position leads to an increase of inhibitory activity toward MetRS and almost complete loss of activity or even activation of LeuRS and vice versa, the fluorine atom (**15**) in this position causes increase of inhibitory activity toward LeuRS and activation of MetRS. Therefore, the order of potency of substituent R^5^ for MetRS could be proposed as following: CF_3_ < Cl < OCH_3_ < CH_3_ < F and for LeuRS as following: F < OCH_3_ < CH_3_ < CF_3_ < Cl. It should be noted, that the R^5^ = methoxy group (compound **20**) is important for membrane permeability since the substitution of OCH_3_ with any other group such as CH_3_, CF_3_, F or Cl leads to complete loss of antibacterial activity toward *M. tuberculosis* resistant strains.

According to structure–activity relationship (SAR) studies of compounds **9–14**, the order of R^6^ substituent inhibitory efficiency for MetRS is following: F < CH_3_CH_2_ < OCH_3_ < H < Cl < Br, and for LeuRS: OCH_3_ < CH_3_CH_2_ < F < H < Cl < Br. For a pair of compounds with R^5^ = OCH_3_ (**20**, **21**), the introduction of R^6^ = methoxy group instead of Hydrogen leads to complete loss, even activation of both LeuRS and MetRS. Moreover, this substitution causes a complete loss of antibacterial activity toward *M. tuberculosis* resistant strain. The nature of the R^7^ substituent for compounds **26**–**29** has a similar effect for both enzymes: Cl < CH_3_ < OCH_3_ < H. The presence of a Cl atom at this position is important for antimycobacterial activity.

Therefore, the chemical structure of all investigated substituents R^1^-R^7^ of 3-Phenyl-5-(1-phenyl-1H-[1,2,3]triazol-4-yl)-[1,2,4]oxadiazole derivatives influence inhibitory activity toward *M.* *tuberculosis* LeuRS and MetRS. Summarizing SAR data of the most active 3-phenyl-5-(1-phenyl-1H-[1,2,3]triazol-4-yl)-[1,2,4]oxadiazole derivatives, we can make some conclusions. So, for dual-targeted inhibitory activity toward *M. tuberculosis* LeuRS and MetRS, the substituents R^4^ = H (compound 2) and R^6^ = OCH_3_ (compounds 13 and 17) are the most profitable. For MetRS inhibitory activity the substituents R^4^ = F (compounds 31 and 34), R^5^ = CF_3_ (compound 19), R^6^ = F (compounds 12, 22, 41), R7 = Cl (compound 27) possess the highest efficiency. For LeuRS inhibitory activity very important role plays fluorine atom as R^5^ substituent (compounds 7 and 15). The established SAR can be useful for further chemical optimization to improve inhibitory activity toward both investigated ARSases as well as antibacterial activity toward *M. tuberculosis* pathogenic strains.

The established SAR of 3-Phenyl-5-(1-phenyl-1H-[1,2,3]triazol-4-yl)-[1,2,4]oxadiazole derivatives can be useful for further chemical optimization to improve inhibitory activity toward *M.* *tuberculosis* LeuRS and MetRS.

**Figure 1:**
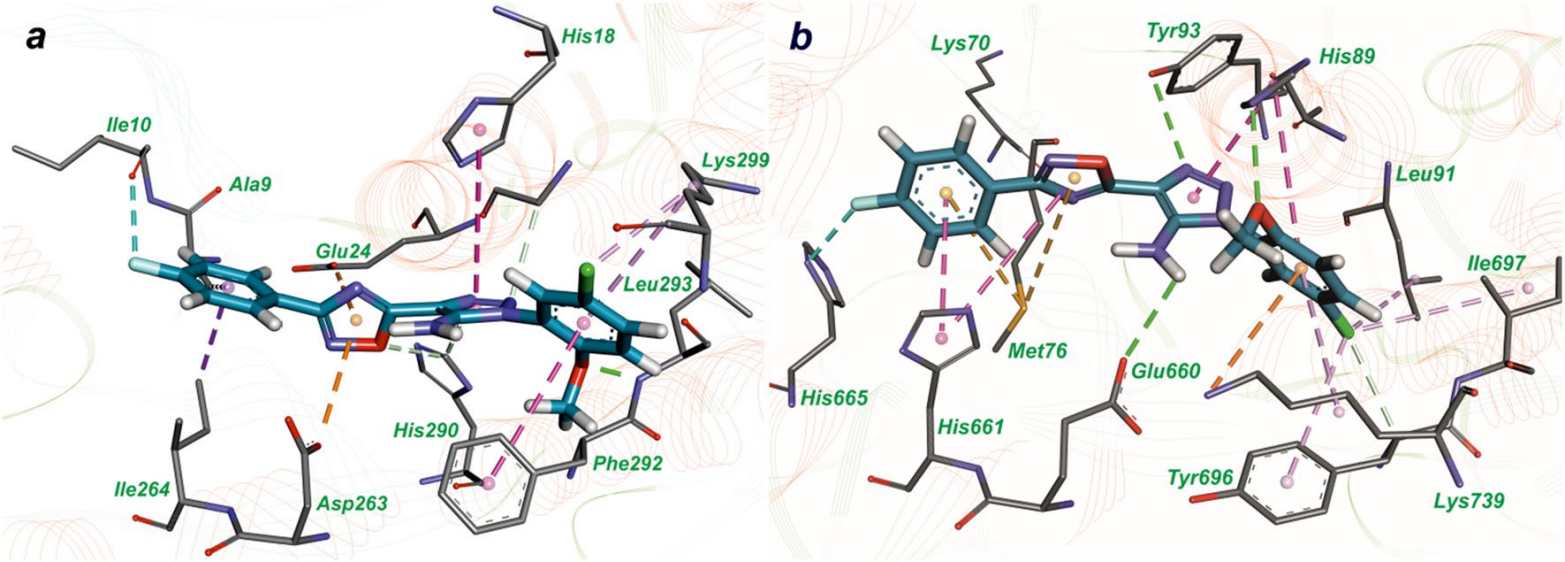
The complexes of compound **27** in the active site of *M. tuberculosis* MetRS (**a**) and LeuRS (**b**), obtained with molecular docking. Hydrogen bonds are shown by the green dotted lines, sulfur–π interaction is presented by the yellow dotted line and hydrophobic interactions are indicated by magenta colors.

Molecular docking results revealed that compounds in this class have similar binding modes with both active sites of *M. tuberculosis* LeuRS and MetRS. The complexes of compound **27** with amino acid residues of MetRS and LeuRS active sites are presented in Fig. 1a and Fig. 1b respectively. Despite compound **27** has better inhibitory activity toward MetRS, than toward LeuRS, binding modes for both enzymes are similar: 2-methoxy-5-chloro-phenyl interacts with the adenine-binding region and 4-fluoro phenyl ring binds to the amino acid binding pocket (Fig. 1).

**Figure 2:**
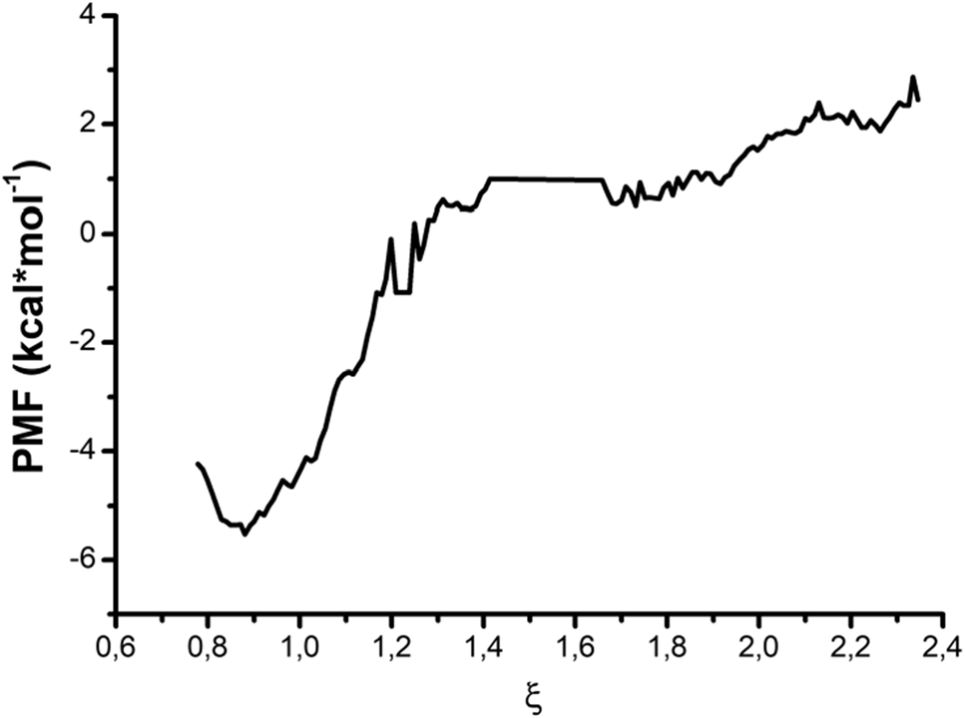
Potential of Mean Force (PMF) for unbinding of the complex of *M. tuberculosis* MetRS with compound **27**.

We have calculated free energy binding (ΔG) for this compound with aminoacyl adenylate binding pocket of mycobacterial MetRS and LeuRS using umbrella sampling algorithm and Weighted Histogram Analysis Method (WHAM). Δ*G*b has been determined from the PMF curve as the difference between the PMF with the ligand-bound minus the ligand when it is unbound (Fig. 2).

According to umbrella sampling calculations, the free energy binding of compound **27** with MetRS is—2 kcal/mol, while for LeuRS it was impossible to build the PMF curve. The profiles of PMF curves for MetRS and LeuRS are available as Supplementary Notes. It seems that the umbrella sampling algorithm is more sensitive in *silico* method to predict the binding affinity of ligands with receptors than docking and can be a useful approach for structure-based optimization of compounds within one chemical class.

## Materials and methods

### Molecular docking

Molecular docking of the ligands into the aminoacyl-adenylate binding sites of MetRS crystal structure (PDB ID: 6AX8) and LeuRS homology model, which was obtained by us earlier^5^, was performed with DOCK 4.0 program^25–28^. The water and ligand molecules were deleted from MetRS PDB-file. Ligand geometry was evaluated in the YFF force field^29^. Partial atomic charges for compounds were set using Kirchhoff method^30^. Docking of the ligands into MetRS and LeuRS active sites was carried out using previously described parameters^21^. The complexes of ligands with MetRS and LeuRS active sites were visually analyzed by Discovery Studio Visualizer^31^.

### Free energy calculation by umbrella sampling

Molecular dynamics simulations were carried out with GROMACS v.4.5^32–34^. The umbrella sampling algorithm and Weighted Histogram Analysis Method (WHAM)^35^ were used to calculate the free energy profile for the separation of MetRS-inhibitor and LeuRS–inhibitor complexes. The starting coordinates used for simulations were from docking complexes.

The topology file for ligand was generated using the web-site Automated Topology Builder (ATB)^36^. Topology files for aminoacyl-tRNA synthetases have been generated from PDB-files using pdb2gmx command. The system was set up using the Gromos96 53a6 force field and solvated with the SPC water model.

The center of mass of the receptor-ligand complex has been placed at (4.0, 4.0, 4.0) in a box of dimensions 12 × 12 × 12 using editconf command. Then, the system was solvated with genbox command and neutralized using Na^+^ or Cl^-^ ions according to the charge of the system using genion command. The periodic boundary conditions and the particle mesh Ewald method were used with a nonbonded cutoff of 9 Å. Each system was first energy minimized using 5000 steps of steepest descent method followed by NPT equilibration for 100 ps. Using the make_ndx command we have defined a custom index group for pulling simulation. The pulling of ligands for each system has been performed in Y-dimension using a force constant of 1000 kJ/(mol nm^2^). A series of configurations along the Y-axis has been generated corresponding to each of the frames saved in the continuous pulling simulation. To measure the distance between protein and ligand on all of these frames, we have used Perl script to iteratively call the g_dist command. The total path with a length of 4.5 nm was divided into 0.1 nm wide equidistant windows. Each coordinate file that is required to obtain 0.1-nm spacing has been prepared for umbrella sampling simulations. At first, NPT equilibration in each window was performed. Then, each input file was passed to the umbrella sampling simulation. Then, using WHAM we have extracted the potential of mean force (PMF), which yields the ΔG for the binding/unbinding process.

The file with a list of commands and MDP files used during umbrella sampling simulation for MetRS-inhibitor and LeuRS–inhibitor complexes are available as Supplementary Methods.

### Purification of recombinant *M. tuberculosis* aminoacyl-tRNA synthetases and in vitro aminoacylation reaction with compounds

The pET28a bacterial expression vector encoding the gene for *M. tuberculosis* LeuRS was a kind gift from Stephen Cusack and Andres Palencia (EMBL, Grenoble). Purification of *M. tuberculosis* LeuRS was performed according to the protocol described previously. The plasmid construct pET28b-MetRS *M. tuberculosis* obtained by our group earlier was used for the expression and purification of the target enzyme. The primary activity of both enzymes and their residual activity after incubation with potential inhibitors were checked in the aminoacylation reaction with Met/Leu and total *E. coli* MRE 600 tRNA (Roche) using the BIOMOL GREEN reagent^21^.

### Growth inhibition determination against *M. tuberculosis* H37Rv and clinically relevant resistant strains

Percent growth inhibition of compounds was determined against *M. tuberculosis* strains using detection of reduction in resazurin (calorimetric growth indicator) compared to untreated growth controls. MIC was determined using optical density (OD) and a calorimetric growth indicator. Compound concentration cutoff values for all test compounds that showed > 80% growth reduction when compared to untreated growth control.

Antibacterial activity of compounds was determined in Middlebrook 7H9 complete medium which was prepared by the following procedure: 4.7 g of 7H9 powder (Difco; Becton Dickinson) was supplemented with 2 mL glycerol (Fisher) and with Milli-Q-water to the final volume 900 mL and mixed until dissolved. To this volume 100 ml of ADC (5 g bovine serum albumin (BSA) (Sigma), 2 g dextrose (Fisher), and 3 mg catalase (Sigma) dissolved in Milli-Q-water to final volume 100 mL) was added. Then, 10 mL of sterile 5% [v:v] polysorbate 80 (tween 80) (Sigma) was added to yield 0.05% [v:v] tween 80 final. Inoculums were prepared with *M. tuberculosis* strains H37Rv, a rifampin-resistant (RIF: *rpoB*^*S450L*^) strain (ATCC #35,838), an isoniazid-resistant (INH: *katG*^*del*^) strain (MS015), a moxifloxacin resistant (MOX: *gyr*^*D94K*^) strain (MOX3), *M. avium* (ATCC 700,891 MAC 101) and *M. abscessus* (ATCC 19,977) strains. The target adjusted final concentration of bacteria was 5 × 10^5^ CFU/mL.

Compounds were dissolved in 100% DMSO. Three drug concentrations were prepared – 20 mg/L, 2 mg/L, and 0.2 mg/L. Assay plates (96-well clear round-bottom plates) were prepared by adding 2 µL of the compound solution to each well at a specific concentration leaving two empty wells untreated growth controls. 100 µL of inoculum was added to all wells. Sealed assay plates were incubated at 37 ºC. On day 7, 10 µL Alamar Blue dye was added to each analytical well. Plates were incubated for 3 days and OD readings were taken at 570 nm and 600 nm. The following formula was used to determine % growth inhibition:

$$  \left( {\frac{{\left[ {\left( {\varepsilon ox} \right)\lambda 2} \right]\left[ {A\lambda 1} \right] - \left[ {\left( {\varepsilon ox} \right)\lambda 1} \right]\left[ {A\lambda 2} \right]of\;test\;agent\;dilution}}{{\left[ {\left( {\varepsilon ox} \right)\lambda 2} \right]\left[ {A\lambda 1} \right] - \left[ {\left( {\varepsilon ox} \right)\lambda 1} \right]\left[ {A\lambda 2} \right]of\;untreated\;positive\;growth\;control}}} \right) \times 100 = Percent\;growth\;reduction,  $$

where λ1 = 570, λ2 = 600, (*ɛox*)λ2 = 117,216, (*ɛox*)λ1 = 80,586.

Minimum inhibitor concentrations (MICs) were established in four Middlebrook 7H9-based media according to the procedure described earlier by us^21^.

#### Cytotoxicity assay

Compound cytotoxicity was determined by standard MTT assay^37^ using human embryonic kidney 293 (HEK293) and human hepatocellular carcinoma (HepG2) cell lines according to the method described previously^38^. HEK 293 cell line was obtained from the Russian Cell Culture Collection (Institute of Cytology of the Russian Academy of Science, St.Petersburg, Russia) and HepG2 – from the Bank of Cell Lines from human and animal tissue (R. E. Kavetsky Institute of Experimental Pathology, Oncology and Radiobiology, NAS of Ukraine, Kyiv, Ukraine).

#### Chemical synthesis

**Figure 3:**
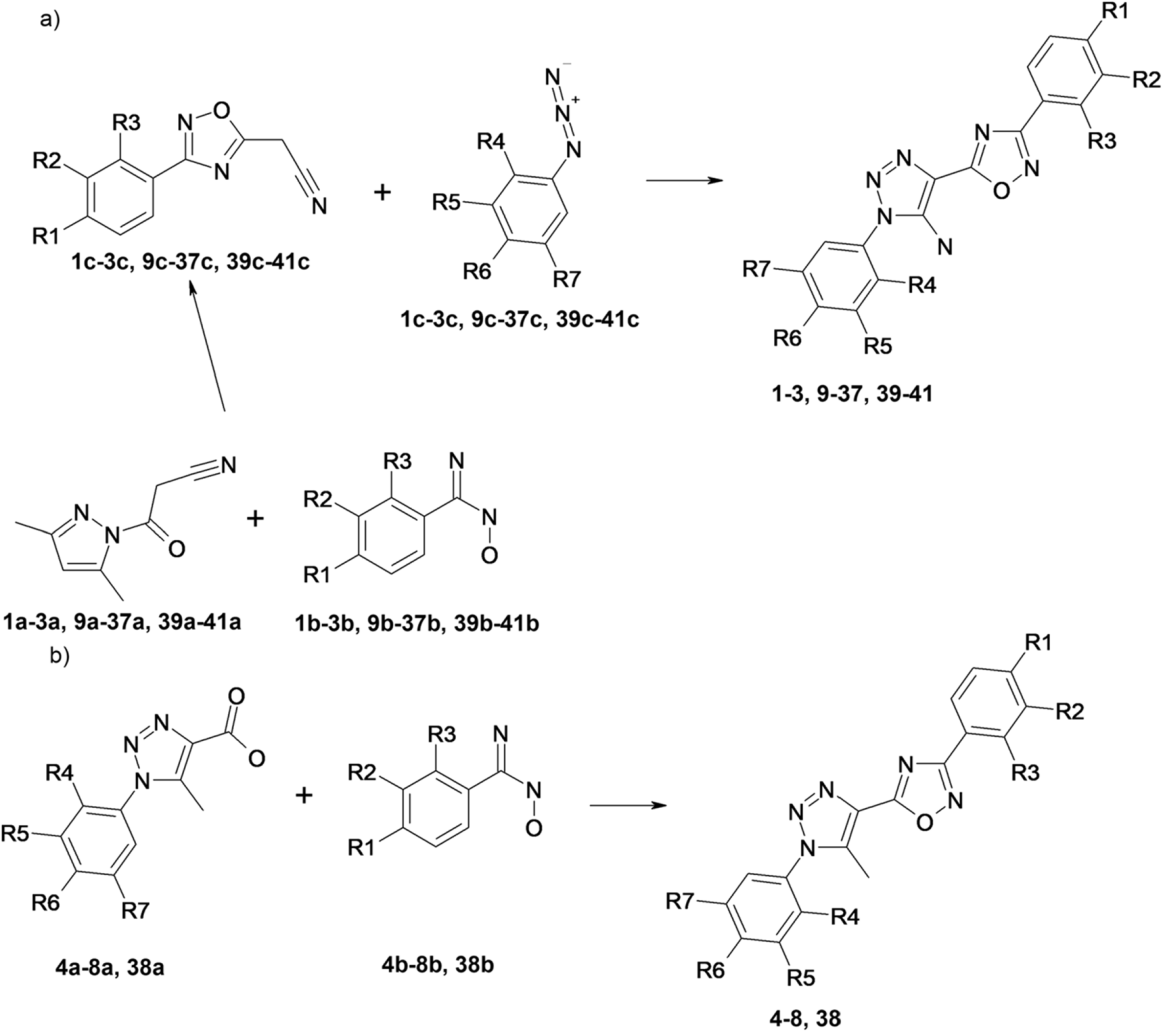
The synthesis pathway for 3-phenyl-5-(1-phenyl-1H-[1,2,3]triazol-4-yl)-[1,2,4]oxadiazole derivatives with R^8^ = NH_2_ (compounds **1–3**, **9–37**, **39–41**) (**a**) and general synthetic procedure for 3-phenyl-5-(1-phenyl-1H-[1,2,3]triazol-4-yl)-[1,2,4]oxadiazole derivatives with R^8^ = CH_3_ (compounds **4–8**, **38**) (**b**).

3-phenyl-5-(1-phenyl-1H-[1,2,3]triazol-4-yl)-[1,2,4]oxadiazole derivatives with R^8^ = NH_2_ (compounds **1–3**, **9–37**, **39–41**) were synthesized as described earlier^39,40^ according to scheme presented in Fig. 3, a. 3-phenyl-5-(1-phenyl-1H-[1,2,3]triazol-4-yl)-[1,2,4]oxadiazole derivatives with R^8^ = CH_3_ (compounds **4–8**, **38**) were synthesized using previously reported method^41^ according to scheme presented in Fig. 3, b.

The structures of the compounds were confirmed using NMR spectroscopy and LC–MS analysis. Nuclear magnetic resonance spectra were recorded on a Varian Mercury VRX-400 spectrometer using DMSO-d6 as solvent and tetramethylsilane as internal standard. Chemical shift values (δ) are quoted in ppm and coupling constants (J) in Hz. Liquid chromatography-mass spectra (LC–MS) analyzes were performed using the Agilent 1100 LC/MSD SL (Agilent Technologies) separations module and Mass Quad G1956B mass detector with electrospray ionization (Agilent Technologies). Spectral data for compounds are presented in Supplementary Methods Section.

## Supplementary Information

Supplementary Information
