# Supplementary material for: Discovery of novel antituberculosis agents among 3-phenyl-5-(1-phenyl-1H-[1,2,3]triazol-4-yl)-[1,2,4]oxadiazole derivatives targeting aminoacyl-tRNA synthetases
Source: Sci Rep. 2021 Mar 30;11:7162. doi: 10.1038/s41598-021-86562-y (PMC8010095; doi:10.1038/s41598-021-86562-y)
Supplement: Supplementary file 1 — Supplementary Information [file 41598_2021_86562_MOESM1_ESM.docx]

**Supplementary Information for**

**Discovery of novel antituberculosis agents among 3-phenyl-5-(1-phenyl-1H-[1,2,3]triazol-4-yl)-[1,2,4]oxadiazole derivatives targeting aminoacyl-tRNA synthetases**

Mariia Yu. Rybak^1*^, Anatoliy O. Balanda^2^, Anna P. Yatsyshyna^3^, Igor. M. Kotey^2^, Sergiy A. Starosyla^2^, Volodymyr G. Bdzhola^2^, Lubov L. Lukash^3^, Sergiy M. Yarmoluk^2^, Michael A. Tukalo^1^, Galyna P. Volynets^2^

^1^ Department of Protein Synthesis Enzymology, Institute of Molecular Biology and Genetics of the NAS of Ukraine, Kyiv, Ukraine

^2^ Department of Medicinal Chemistry, Institute of Molecular Biology and Genetics of the NAS of Ukraine, Kyiv, Ukraine

^3^ Department of Human Genetics, Institute of Molecular Biology and Genetics of the NAS of Ukraine, Kyiv, Ukraine

^*^Corresponding author

E-mail: [mariia.rybak@gmail.com](mailto:mariia.rybak@gmail.com)

**Content**

**Supplementary Methods**

List of commands for LeuRS (umbrella sampling)

List of commands for MetRS (umbrella sampling)

Characteristics of compounds (^1^H NMR, ^13^C NMR and LS-MS spectra)

**Supplementary Notes**

Potential of Mean Force (PMF) profile for LeuRS

Potential of Mean Force (PMF) for MetRS

**Supplementary Methods**

**List of commands for LeuRS (umbrella sampling)**

#pdb2gmx -f p.pdb -o p.gro -p p.top

#editconf -f p.gro -o newbox.gro -center 4 4 4 -box 12 12 12

#genbox -cp newbox.gro -cs -o solv.gro -p p.top

#grompp -f ions.mdp -c solv.gro -p p.top -o ions.tpr -maxwarn 5

#genion -s ions.tpr -o solv_ions.gro -pname Na -pq 0 -np 35

#grompp -f minim.mdp -c solv_ions.gro -p p.top -o em.tpr

#mdrun -v -deffnm em

#grompp -f npt.mdp -c em.gro -p p.top -o npt.tpr -maxwarn 5

#nohup mdrun -deffnm npt

#make_ndx -f npt.gro

#grompp -f md_pull.mdp -c npt.gro -p p.top -n index.ndx -t npt.cpt -o pull.tpr

#nohup mdrun -s pull.tpr

#trjconv -s pull.tpr -f traj.xtc -o conf.gro -sep

#g_dist -s pull.tpr -f conf9.gro -n index.ndx -o dist9.xvg

#grompp -f npt_umbrella.mdp -c conf1.gro -p p.top -n index.ndx -o npt1.tpr -maxwarn 5

#mdrun -deffnm npt1

#grompp -f npt_umbrella.mdp -c conf4.gro -p p.top -n index.ndx -o npt4.tpr -maxwarn 5

#mdrun -deffnm npt4

#grompp -f npt_umbrella.mdp -c conf57.gro -p p.top -n index.ndx -o npt57.tpr -maxwarn 5

#mdrun -deffnm npt57

#grompp -f npt_umbrella.mdp -c conf70.gro -p p.top -n index.ndx -o npt70.tpr -maxwarn 5

#mdrun -deffnm npt70

#grompp -f npt_umbrella.mdp -c conf81.gro -p p.top -n index.ndx -o npt81.tpr -maxwarn 5

#mdrun -deffnm npt81

#grompp -f npt_umbrella.mdp -c conf102.gro -p p.top -n index.ndx -o npt102.tpr -maxwarn 5

#mdrun -deffnm npt102

#grompp -f npt_umbrella.mdp -c conf108.gro -p p.top -n index.ndx -o npt108.tpr -maxwarn 5

#mdrun -deffnm npt108

#grompp -f npt_umbrella.mdp -c conf120.gro -p p.top -n index.ndx -o npt120.tpr -maxwarn 5

#mdrun -deffnm npt120

#grompp -f npt_umbrella.mdp -c conf125.gro -p p.top -n index.ndx -o npt125.tpr -maxwarn 5

#mdrun -deffnm npt125

#grompp -f npt_umbrella.mdp -c conf134.gro -p p.top -n index.ndx -o npt134.tpr -maxwarn 5

#mdrun -deffnm npt134

#grompp -f npt_umbrella.mdp -c conf141.gro -p p.top -n index.ndx -o npt141.tpr -maxwarn 5

#mdrun -deffnm npt141

#grompp -f npt_umbrella.mdp -c conf144.gro -p p.top -n index.ndx -o npt144.tpr -maxwarn 5

#mdrun -deffnm npt144

#grompp -f npt_umbrella.mdp -c conf150.gro -p p.top -n index.ndx -o npt150.tpr -maxwarn 5

#mdrun -deffnm npt150

#grompp -f npt_umbrella.mdp -c conf156.gro -p p.top -n index.ndx -o npt156.tpr -maxwarn 5

#mdrun -deffnm npt156

#grompp -f npt_umbrella.mdp -c conf168.gro -p p.top -n index.ndx -o npt168.tpr -maxwarn 5

#mdrun -deffnm npt168

#grompp -f npt_umbrella.mdp -c conf168.gro -p p.top -n index.ndx -o npt168.tpr -maxwarn 5

#mdrun -deffnm npt168

#grompp -f npt_umbrella.mdp -c conf186.gro -p p.top -n index.ndx -o npt186.tpr -maxwarn 5

#mdrun -deffnm npt186

#grompp -f npt_umbrella.mdp -c conf199.gro -p p.top -n index.ndx -o npt199.tpr -maxwarn 5

#mdrun -deffnm npt199

#grompp -f npt_umbrella.mdp -c conf202.gro -p p.top -n index.ndx -o npt202.tpr -maxwarn 5

#mdrun -deffnm npt202

#grompp -f npt_umbrella.mdp -c conf207.gro -p p.top -n index.ndx -o npt207.tpr -maxwarn 5

#mdrun -deffnm npt207

#grompp -f npt_umbrella.mdp -c conf216.gro -p p.top -n index.ndx -o npt216.tpr -maxwarn 5

#mdrun -deffnm npt216

#grompp -f npt_umbrella.mdp -c conf228.gro -p p.top -n index.ndx -o npt228.tpr -maxwarn 5

#mdrun -deffnm npt228

#grompp -f npt_umbrella.mdp -c conf232.gro -p p.top -n index.ndx -o npt232.tpr -maxwarn 5

#mdrun -deffnm npt232

#grompp -f npt_umbrella.mdp -c conf251.gro -p p.top -n index.ndx -o npt251.tpr -maxwarn 5

#mdrun -deffnm npt251

#grompp -f npt_umbrella.mdp -c conf256.gro -p p.top -n index.ndx -o npt256.tpr -maxwarn 5

#mdrun -deffnm npt256

#grompp -f npt_umbrella.mdp -c conf262.gro -p p.top -n index.ndx -o npt262.tpr -maxwarn 5

#mdrun -deffnm npt262

#grompp -f npt_umbrella.mdp -c conf276.gro -p p.top -n index.ndx -o npt276.tpr -maxwarn 5

#mdrun -deffnm npt276

#grompp -f npt_umbrella.mdp -c conf286.gro -p p.top -n index.ndx -o npt286.tpr -maxwarn 5

#mdrun -deffnm npt286

#grompp -f npt_umbrella.mdp -c conf295.gro -p p.top -n index.ndx -o npt295.tpr -maxwarn 5

#mdrun -deffnm npt295

#grompp -f md_umbrella.mdp -c npt1.gro -t npt1.cpt -p p.top -n index.ndx -o umbrella1.tpr

#mdrun -deffnm umbrella1 -pf pullf-umbrella1.xvg -px pullx-umbrella1.xvg

#grompp -f md_umbrella.mdp -c npt4.gro -t npt4.cpt -p p.top -n index.ndx -o umbrella4.tpr

#mdrun -deffnm umbrella4 -pf pullf-umbrella4.xvg -px pullx-umbrella4.xvg

#grompp -f md_umbrella.mdp -c npt57.gro -t npt57.cpt -p p.top -n index.ndx -o umbrella57.tpr

#mdrun -deffnm umbrella57 -pf pullf-umbrella57.xvg -px pullx-umbrella57.xvg

#grompp -f md_umbrella.mdp -c npt70.gro -t npt70.cpt -p p.top -n index.ndx -o umbrella70.tpr

#mdrun -deffnm umbrella70 -pf pullf-umbrella70.xvg -px pullx-umbrella70.xvg

#grompp -f md_umbrella.mdp -c npt81.gro -t npt81.cpt -p p.top -n index.ndx -o umbrella81.tpr

#mdrun -deffnm umbrella81 -pf pullf-umbrella81.xvg -px pullx-umbrella81.xvg

#grompp -f md_umbrella.mdp -c npt102.gro -t npt102.cpt -p p.top -n index.ndx -o umbrella102.tpr

#mdrun -deffnm umbrella102 -pf pullf-umbrella102.xvg -px pullx-umbrella102.xvg

#grompp -f md_umbrella.mdp -c npt108.gro -t npt108.cpt -p p.top -n index.ndx -o umbrella108.tpr

#mdrun -deffnm umbrella108 -pf pullf-umbrella108.xvg -px pullx-umbrella108.xvg

#grompp -f md_umbrella.mdp -c npt120.gro -t npt120.cpt -p p.top -n index.ndx -o umbrella120.tpr

#mdrun -deffnm umbrella120 -pf pullf-umbrella120.xvg -px pullx-umbrella120.xvg

#grompp -f md_umbrella.mdp -c npt125.gro -t npt125.cpt -p p.top -n index.ndx -o umbrella125.tpr

#mdrun -deffnm umbrella125 -pf pullf-umbrella125.xvg -px pullx-umbrella125.xvg

#grompp -f md_umbrella.mdp -c npt134.gro -t npt134.cpt -p p.top -n index.ndx -o umbrella134.tpr

#mdrun -deffnm umbrella134 -pf pullf-umbrella134.xvg -px pullx-umbrella134.xvg

#grompp -f md_umbrella.mdp -c npt144.gro -t npt144.cpt -p p.top -n index.ndx -o umbrella144.tpr

#mdrun -deffnm umbrella144 -pf pullf-umbrella144.xvg -px pullx-umbrella144.xvg

#grompp -f md_umbrella.mdp -c npt150.gro -t npt150.cpt -p p.top -n index.ndx -o umbrella150.tpr

#mdrun -deffnm umbrella150 -pf pullf-umbrella150.xvg -px pullx-umbrella150.xvg

#grompp -f md_umbrella.mdp -c npt156.gro -t npt156.cpt -p p.top -n index.ndx -o umbrella156.tpr

#mdrun -deffnm umbrella156 -pf pullf-umbrella156.xvg -px pullx-umbrella156.xvg

#grompp -f md_umbrella.mdp -c npt168.gro -t npt168.cpt -p p.top -n index.ndx -o umbrella168.tpr

#mdrun -deffnm umbrella168 -pf pullf-umbrella168.xvg -px pullx-umbrella168.xvg

#grompp -f md_umbrella.mdp -c npt186.gro -t npt186.cpt -p p.top -n index.ndx -o umbrella186.tpr

#mdrun -deffnm umbrella186 -pf pullf-umbrella186.xvg -px pullx-umbrella186.xvg

#grompp -f md_umbrella.mdp -c npt199.gro -t npt199.cpt -p p.top -n index.ndx -o umbrella199.tpr

#mdrun -deffnm umbrella199 -pf pullf-umbrella199.xvg -px pullx-umbrella199.xvg

#grompp -f md_umbrella.mdp -c npt202.gro -t npt202.cpt -p p.top -n index.ndx -o umbrella202.tpr

#mdrun -deffnm umbrella202 -pf pullf-umbrella202.xvg -px pullx-umbrella202.xvg

#grompp -f md_umbrella.mdp -c npt207.gro -t npt207.cpt -p p.top -n index.ndx -o umbrella207.tpr

#mdrun -deffnm umbrella207 -pf pullf-umbrella207.xvg -px pullx-umbrella207.xvg

#grompp -f md_umbrella.mdp -c npt216.gro -t npt216.cpt -p p.top -n index.ndx -o umbrella216.tpr

#mdrun -deffnm umbrella216 -pf pullf-umbrella216.xvg -px pullx-umbrella216.xvg

#grompp -f md_umbrella.mdp -c npt228.gro -t npt228.cpt -p p.top -n index.ndx -o umbrella228.tpr

#mdrun -deffnm umbrella228 -pf pullf-umbrella228.xvg -px pullx-umbrella228.xvg

#grompp -f md_umbrella.mdp -c npt232.gro -t npt232.cpt -p p.top -n index.ndx -o umbrella232.tpr

#mdrun -deffnm umbrella232 -pf pullf-umbrella232.xvg -px pullx-umbrella232.xvg

#grompp -f md_umbrella.mdp -c npt251.gro -t npt251.cpt -p p.top -n index.ndx -o umbrella251.tpr

#mdrun -deffnm umbrella251 -pf pullf-umbrella251.xvg -px pullx-umbrella251.xvg

#grompp -f md_umbrella.mdp -c npt256.gro -t npt256.cpt -p p.top -n index.ndx -o umbrella256.tpr

#mdrun -deffnm umbrella256 -pf pullf-umbrella256.xvg -px pullx-umbrella256.xvg

#grompp -f md_umbrella.mdp -c npt262.gro -t npt262.cpt -p p.top -n index.ndx -o umbrella262.tpr

#mdrun -deffnm umbrella262 -pf pullf-umbrella262.xvg -px pullx-umbrella262.xvg

#grompp -f md_umbrella.mdp -c npt276.gro -t npt276.cpt -p p.top -n index.ndx -o umbrella276.tpr

#mdrun -deffnm umbrella276 -pf pullf-umbrella276.xvg -px pullx-umbrella276.xvg

#grompp -f md_umbrella.mdp -c npt286.gro -t npt286.cpt -p p.top -n index.ndx -o umbrella286.tpr

#mdrun -deffnm umbrella286 -pf pullf-umbrella286.xvg -px pullx-umbrella286.xvg

#grompp -f md_umbrella.mdp -c npt295.gro -t npt295.cpt -p p.top -n index.ndx -o umbrella295.tpr

#mdrun -deffnm umbrella295 -pf pullf-umbrella295.xvg -px pullx-umbrella295.xvg

g_wham -it tpr-files.dat -if pullf-files.dat -o -hist -unit kCal

**Supplementary Methods**

**List of commands for MetRS (umbrella sampling)**

#pdb2gmx -f p.pdb -o p.gro -p p.top

#editconf -f p.gro -o newbox.gro -center 4 4 4 -box 12 12 12

#genbox -cp newbox.gro -cs -o solv.gro -p p.top

#grompp -f ions.mdp -c solv.gro -p p.top -o ions.tpr

#genion -s ions.tpr -o solv_ions.gro -pname Na -pq 1 -np 15

#grompp -f minim.mdp -c solv_ions.gro -p p.top -o em.tpr -maxwarn 5

#mdrun -v -deffnm em

#grompp -f npt.mdp -c em.gro -p p.top -o npt.tpr

#nohup mdrun -deffnm npt

#make_ndx -f npt.gro

#grompp -f md_pull.mdp -c npt.gro -p p.top -n index.ndx -t npt.cpt -o pull.tpr

#nohup mdrun -s pull.tpr

#trjconv -s pull.tpr -f traj.xtc -o conf.gro -sep

#g_dist -s pull.tpr -f conf1.gro -n index.ndx -o dist1.xvg

#grompp -f npt_umbrella.mdp -c conf1.gro -p p.top -n index.ndx -o npt1.tpr -maxwarn 5

#mdrun -deffnm npt1

#grompp -f npt_umbrella.mdp -c conf3.gro -p p.top -n index.ndx -o npt3.tpr -maxwarn 5

#mdrun -deffnm npt3

#grompp -f npt_umbrella.mdp -c conf5.gro -p p.top -n index.ndx -o npt5.tpr -maxwarn 5

#mdrun -deffnm npt5

#grompp -f npt_umbrella.mdp -c conf12.gro -p p.top -n index.ndx -o npt12.tpr -maxwarn 5

#mdrun -deffnm npt12

#grompp -f npt_umbrella.mdp -c conf25.gro -p p.top -n index.ndx -o npt25.tpr -maxwarn 5

#mdrun -deffnm npt25

#grompp -f npt_umbrella.mdp -c conf90.gro -p p.top -n index.ndx -o npt90.tpr -maxwarn 5

#mdrun -deffnm npt90

#grompp -f npt_umbrella.mdp -c conf97.gro -p p.top -n index.ndx -o npt97.tpr -maxwarn 5

#mdrun -deffnm npt97

#grompp -f npt_umbrella.mdp -c conf101.gro -p p.top -n index.ndx -o npt101.tpr -maxwarn 5

#mdrun -deffnm npt101

#grompp -f npt_umbrella.mdp -c conf102.gro -p p.top -n index.ndx -o npt102.tpr -maxwarn 5

#mdrun -deffnm npt102

#grompp -f npt_umbrella.mdp -c conf106.gro -p p.top -n index.ndx -o npt106.tpr -maxwarn 5

#mdrun -deffnm npt106

#grompp -f npt_umbrella.mdp -c conf109.gro -p p.top -n index.ndx -o npt109.tpr -maxwarn 5

#mdrun -deffnm npt109

#grompp -f npt_umbrella.mdp -c conf118.gro -p p.top -n index.ndx -o npt118.tpr -maxwarn 5

#mdrun -deffnm npt118

#grompp -f npt_umbrella.mdp -c conf119.gro -p p.top -n index.ndx -o npt119.tpr -maxwarn 5

#mdrun -deffnm npt119

#grompp -f npt_umbrella.mdp -c conf120.gro -p p.top -n index.ndx -o npt120.tpr -maxwarn 5

#mdrun -deffnm npt120

#grompp -f npt_umbrella.mdp -c conf128.gro -p p.top -n index.ndx -o npt128.tpr -maxwarn 5

#mdrun -deffnm npt128

#grompp -f npt_umbrella.mdp -c conf154.gro -p p.top -n index.ndx -o npt154.tpr -maxwarn 5

#mdrun -deffnm npt154

#grompp -f npt_umbrella.mdp -c conf166.gro -p p.top -n index.ndx -o npt166.tpr -maxwarn 5

#mdrun -deffnm npt166

#grompp -f npt_umbrella.mdp -c conf169.gro -p p.top -n index.ndx -o npt169.tpr -maxwarn 5

#mdrun -deffnm npt169

#grompp -f npt_umbrella.mdp -c conf170.gro -p p.top -n index.ndx -o npt170.tpr -maxwarn 5

#mdrun -deffnm npt170

#grompp -f npt_umbrella.mdp -c conf170.gro -p p.top -n index.ndx -o npt170.tpr -maxwarn 5

#mdrun -deffnm npt170

#grompp -f npt_umbrella.mdp -c conf176.gro -p p.top -n index.ndx -o npt176.tpr -maxwarn 5

#mdrun -deffnm npt176

#grompp -f npt_umbrella.mdp -c conf177.gro -p p.top -n index.ndx -o npt177.tpr -maxwarn 5

#mdrun -deffnm npt177

#grompp -f npt_umbrella.mdp -c conf182.gro -p p.top -n index.ndx -o npt182.tpr -maxwarn 5

#mdrun -deffnm npt182

#grompp -f npt_umbrella.mdp -c conf187.gro -p p.top -n index.ndx -o npt187.tpr -maxwarn 5

#mdrun -deffnm npt187

#grompp -f npt_umbrella.mdp -c conf194.gro -p p.top -n index.ndx -o npt194.tpr -maxwarn 5

#mdrun -deffnm npt194

#grompp -f md_umbrella.mdp -c npt1.gro -t npt1.cpt -p p.top -n index.ndx -o umbrella1.tpr

#mdrun -deffnm umbrella1 -pf pullf-umbrella1.xvg -px pullx-umbrella1.xvg

#grompp -f md_umbrella.mdp -c npt3.gro -t npt3.cpt -p p.top -n index.ndx -o umbrella3.tpr

#mdrun -deffnm umbrella3 -pf pullf-umbrella3.xvg -px pullx-umbrella3.xvg

#grompp -f md_umbrella.mdp -c npt5.gro -t npt5.cpt -p p.top -n index.ndx -o umbrella5.tpr

#mdrun -deffnm umbrella5 -pf pullf-umbrella5.xvg -px pullx-umbrella5.xvg

#grompp -f md_umbrella.mdp -c npt12.gro -t npt12.cpt -p p.top -n index.ndx -o umbrella12.tpr

#mdrun -deffnm umbrella12 -pf pullf-umbrella12.xvg -px pullx-umbrella12.xvg

#grompp -f md_umbrella.mdp -c npt25.gro -t npt25.cpt -p p.top -n index.ndx -o umbrella25.tpr

#mdrun -deffnm umbrella25 -pf pullf-umbrella25.xvg -px pullx-umbrella25.xvg

#grompp -f md_umbrella.mdp -c npt90.gro -t npt90.cpt -p p.top -n index.ndx -o umbrella90.tpr

#mdrun -deffnm umbrella90 -pf pullf-umbrella90.xvg -px pullx-umbrella90.xvg

#grompp -f md_umbrella.mdp -c npt97.gro -t npt97.cpt -p p.top -n index.ndx -o umbrella97.tpr

#mdrun -deffnm umbrella97 -pf pullf-umbrella97.xvg -px pullx-umbrella97.xvg

#grompp -f md_umbrella.mdp -c npt101.gro -t npt101.cpt -p p.top -n index.ndx -o umbrella101.tpr

#mdrun -deffnm umbrella101 -pf pullf-umbrella101.xvg -px pullx-umbrella101.xvg

#grompp -f md_umbrella.mdp -c npt102.gro -t npt102.cpt -p p.top -n index.ndx -o umbrella102.tpr

#mdrun -deffnm umbrella102 -pf pullf-umbrella102.xvg -px pullx-umbrella102.xvg

#grompp -f md_umbrella.mdp -c npt106.gro -t npt106.cpt -p p.top -n index.ndx -o umbrella106.tpr

#mdrun -deffnm umbrella106 -pf pullf-umbrella106.xvg -px pullx-umbrella106.xvg

#grompp -f md_umbrella.mdp -c npt109.gro -t npt109.cpt -p p.top -n index.ndx -o umbrella109.tpr

#mdrun -deffnm umbrella109 -pf pullf-umbrella109.xvg -px pullx-umbrella109.xvg

#grompp -f md_umbrella.mdp -c npt118.gro -t npt118.cpt -p p.top -n index.ndx -o umbrella118.tpr

#mdrun -deffnm umbrella118 -pf pullf-umbrella118.xvg -px pullx-umbrella118.xvg

#grompp -f md_umbrella.mdp -c npt119.gro -t npt119.cpt -p p.top -n index.ndx -o umbrella119.tpr

#mdrun -deffnm umbrella119 -pf pullf-umbrella119.xvg -px pullx-umbrella119.xvg

#grompp -f md_umbrella.mdp -c npt120.gro -t npt120.cpt -p p.top -n index.ndx -o umbrella120.tpr

#mdrun -deffnm umbrella120 -pf pullf-umbrella120.xvg -px pullx-umbrella120.xvg

#grompp -f md_umbrella.mdp -c npt128.gro -t npt128.cpt -p p.top -n index.ndx -o umbrella128.tpr

#mdrun -deffnm umbrella128 -pf pullf-umbrella128.xvg -px pullx-umbrella128.xvg

#grompp -f md_umbrella.mdp -c npt154.gro -t npt154.cpt -p p.top -n index.ndx -o umbrella154.tpr

#mdrun -deffnm umbrella154 -pf pullf-umbrella154.xvg -px pullx-umbrella154.xvg

#grompp -f md_umbrella.mdp -c npt166.gro -t npt166.cpt -p p.top -n index.ndx -o umbrella166.tpr

#mdrun -deffnm umbrella166 -pf pullf-umbrella166.xvg -px pullx-umbrella166.xvg

#grompp -f md_umbrella.mdp -c npt169.gro -t npt169.cpt -p p.top -n index.ndx -o umbrella169.tpr

#mdrun -deffnm umbrella169 -pf pullf-umbrella169.xvg -px pullx-umbrella169.xvg

#grompp -f md_umbrella.mdp -c npt170.gro -t npt170.cpt -p p.top -n index.ndx -o umbrella170.tpr

#mdrun -deffnm umbrella170 -pf pullf-umbrella170.xvg -px pullx-umbrella170.xvg

#grompp -f md_umbrella.mdp -c npt176.gro -t npt176.cpt -p p.top -n index.ndx -o umbrella176.tpr

#mdrun -deffnm umbrella176 -pf pullf-umbrella176.xvg -px pullx-umbrella176.xvg

#grompp -f md_umbrella.mdp -c npt177.gro -t npt177.cpt -p p.top -n index.ndx -o umbrella177.tpr

#mdrun -deffnm umbrella177 -pf pullf-umbrella177.xvg -px pullx-umbrella177.xvg

#grompp -f md_umbrella.mdp -c npt182.gro -t npt182.cpt -p p.top -n index.ndx -o umbrella182.tpr

#mdrun -deffnm umbrella182 -pf pullf-umbrella182.xvg -px pullx-umbrella182.xvg

#grompp -f md_umbrella.mdp -c npt187.gro -t npt187.cpt -p p.top -n index.ndx -o umbrella187.tpr

#mdrun -deffnm umbrella187 -pf pullf-umbrella187.xvg -px pullx-umbrella187.xvg

#grompp -f md_umbrella.mdp -c npt194.gro -t npt194.cpt -p p.top -n index.ndx -o umbrella194.tpr

#mdrun -deffnm umbrella194 -pf pullf-umbrella194.xvg -px pullx-umbrella194.xvg

g_wham -it tpr-files.dat -if pullf-files.dat -o -hist -unit kCal

**Supplementary Methods**

**Characteristics of compounds (**^1^H NMR, ^13^C NMR and LS-MS spectra**)**

Compound **1** (ID: 1032252) *1-(2-fluorophenyl)-4-(3-phenyl-1,2,4-oxadiazol-5-yl)-1H-1,2,3-triazol-5-amine*: Yield 85%. 1H NMR (400 MHz, DMSO-d6) δ 8.21 (d, J = 6.3 Hz, 2H), 7.70 (q, J = 7.3 Hz, 2H), 7.63 – 7.52 (m, 4H), 7.46 (t, J = 7.7 Hz, 1H), 7.14 (s, 2H). 13C NMR (101 MHz, DMSO-d6) δ 169.40, 167.75, 158.37, 155.86, 146.33, 133.25, 133.23, 132.03, 129.83, 129.54, 127.88, 126.64, 126.04, 126.03, 122.19, 117.83, 117.74, 117.54, 114.23. LC-MS: m/z 323 [M+H]+.

Compound **2** (ID: 1032254) *1-[3-chloro-4-(trifluoromethyl)phenyl]-4-(3-phenyl-1,2,4-oxadiazol-5-yl)-1H-1,2,3-triazol-5-amine*: Yield 81%. ^1^H NMR (400 MHz, DMSO-*d*_6_) δ 8.25 – 8.06 (m, 3H), 8.06 – 7.90 (m, 2H), 7.67 – 7.38 (m, 3H), 7.10 (s, 2H). ^13^C NMR (101 MHz, DMSO-*d*_6_) δ 169.37, 167.76, 145.63, 133.88, 133.61, 132.24, 132.02, 131.34, 129.53, 128.49, 127.85, 126.59, 125.46, 122.30, 115.08, 45.37. LC-MS: *m/z* 407 [M+H]^+^.

Compound **3** (ID: 1032257) *1-[3-chloro-4-(trifluoromethyl)phenyl]-4-[3-(2-fluorophenyl)-1,2,4-oxadiazol-5-yl]-1H-1,2,3-triazol-5-amine*: Yield 84%. ^1^H NMR (400 MHz, DMSO-*d*_6_) δ 8.36 (t, *J* = 7.6 Hz, 1H), 8.14 (s, 1H), 8.00 (s, 2H), 7.71 – 7.55 (m, 1H), 7.51 – 7.30 (m, 2H), 7.16 (s, 2H). ^13^C NMR (101 MHz, DMSO-*d*_6_) δ 168.79, 164.63, 161.68, 159.13, 145.68, 134.02, 133.86, 133.63, 132.28, 131.81, 131.37, 128.51, 128.20, 125.46, 124.06, 121.35, 117.23, 117.02, 114.93, 114.74. LC-MS: *m/z* 425 [M+H]^+^.

Compound **4** (ID: 1030196) *5-[1-(3-chloro-4-fluorophenyl)-5-methyl-1H-1,2,3-triazol-4-yl]-3-(2-fluorophenyl)-1,2,4-oxadiazole:* Yield 68%. ^1^H NMR (400 MHz, DMSO-*d*_6_) δ 8.16-8.07 (m, 2H), 7.84 – 7.72 (m, 2H), 7.68 (q, *J* = 6.4 Hz, 1H), 7.52 – 7.40 (m, 2H), 2.70 (s, 3H). ^13^C NMR (101 MHz, DMSO-*d*_6_) δ 169.15, 165.16, 161.62, 159.81, 159.08, 157.32, 139.39, 134.33, 134.25, 132.30, 132.26, 131.43, 131.12, 128.56, 128.51, 127.33, 127.31, 127.25, 125.69, 125.66, 121.32, 118.68, 118.46, 117.47, 117.26, 114.48, 114.36, 10.10. LC-MS: *m/z* 374 [M+H]^+^.

Compound **5** (ID: 1032348) *5-[1-(4-bromophenyl)-5-methyl-1H-1,2,3-triazol-4-yl]-3-(2-fluorophenyl)-1,2,4-oxadiazole:* Yield 72%. ^1^H NMR (400 MHz, DMSO-*d*_6_) δ 8.11 (t, *J* = 7.6 Hz, 1H), 7.87 (d, *J* = 7.8 Hz, 2H), 7.66 (dd, *J* = 14.7, 5.7 Hz, 3H), 7.50 – 7.35 (m, 2H), 2.69 (s, 3H). ^13^C NMR (101 MHz, DMSO-*d*_6_) δ 169.18, 165.17, 165.11, 161.62, 159.08, 138.95, 134.62, 134.26, 134.17, 133.28, 131.60, 131.10, 127.86, 125.63, 125.60, 124.07, 117.44, 117.41, 117.23, 114.50, 114.37, 10.20. LC-MS: *m/z* 401 [M+H]^+^.

Compound 7 (ID: 1032359) *3-(2-chlorophenyl)-5-[1-(3-fluorophenyl)-5-methyl-1H-1,2,3-triazol-4-yl]-1,2,4-oxadiazole:* Yield 76%. ^1^H NMR (400 MHz, DMSO-*d*_6_) δ 8.04 (dd, *J* = 7.6, 1.8 Hz, 1H), 7.78 – 7.70 (m, 3H), 7.68 – 7.51 (m, 4H), 2.73 (s, 3H). ^13^C NMR (101 MHz, DMSO-*d*_6_) δ 168.70, 166.77, 163.22, 160.77, 138.63, 136.15, 136.05, 132.77, 132.22, 131.80, 131.73, 131.64, 131.07, 130.88, 127.73, 125.12, 121.79, 121.76, 117.51, 117.30, 113.22, 112.97, 9.72. LC-MS: *m/z* 356 [M+H]^+^.

Compound **9** (ID: 1032263) *4-[3-(4-fluorophenyl)-1,2,4-oxadiazol-5-yl]-1-phenyl-1H-1,2,3-triazol-5-amine:*Yield 85%. ^1^H NMR (400 MHz, DMSO-*d*_6_) δ 8.37 – 8.12 (m, 2H), 7.75 – 7.49 (m, 5H), 7.48 – 7.27 (m, 2H), 6.95 (s, 2H). ^13^C NMR (101 MHz, DMSO-*d*_6_) δ 169.54, 166.94, 163.21, 145.15, 134.80, 130.35, 130.03, 125.20, 123.22, 116.76, 116.54, 115.14. LC-MS: *m/z* 323 [M+H]^+^.

Compound **11** (ID: 1032292) *1-(4-bromophenyl)-4-[3-(4-fluorophenyl)-1,2,4-oxadiazol-5-yl]-1H-1,2,3-triazol-5-amine:* Yield 87%. ^1^H NMR (400 MHz, DMSO-*d*_6_) δ 8.22 (m, 2H), 7.80 (d, *J* = 8.6 Hz, 2H), 7.60 (d, *J* = 8.6 Hz, 2H), 7.31 (2H t, *J* = 8.7 Hz, 2H), 6.79 (s, 2H). ^13^C NMR (101 MHz, DMSO-*d*_6_) δ 169.02, 166.51, 165.24, 162.77, 144.87, 133.54, 132.81, 132.70, 130.03, 129.94, 127.11, 122.76, 122.71, 121.30, 116.34, 116.12, 114.60, 40.44. LC-MS: *m/z* 401 [M+H]^+^.

Compound **12** (ID: 1032268) *1-(4-fluorophenyl)-4-[3-(4-fluorophenyl)-1,2,4-oxadiazol-5-yl]-1H-1,2,3-triazol-5-amine:* Yield 83%. ^1^H NMR (400 MHz, DMSO-*d*_6_) δ 8.33 – 8.23 (m, 2H), 7.76 – 7.66 (m, 2H), 7.51 (t, *J* = 8.8 Hz, 2H), 7.42 (t, *J* = 8.8 Hz, 2H), 6.99 (s, 2H). ^13^C NMR (101 MHz, DMSO-*d*_6_) δ 169.07, 166.49, 165.23, 163.57, 162.75, 161.12, 145.01, 130.63, 130.60, 130.01, 129.92, 127.76, 127.67, 122.77, 122.73, 116.87, 116.64, 116.32, 116.10, 114.44. LC-MS: *m/z* 341 [M+H]^+^.

Compound **13** (ID: 1032277) *4-[3-(4-fluorophenyl)-1,2,4-oxadiazol-5-yl]-1-(4-methoxyphenyl)-1H-1,2,3-triazol-5-amine:* Yield 80%. ^1^H NMR (400 MHz, DMSO-*d*_6_) δ 8.33 – 8.18 (m, 2H), 7.55 (d, *J* = 8.9 Hz, 2H), 7.42 (t, *J* = 8.8 Hz, 2H), 7.19 (d, *J* = 8.9 Hz, 2H), 6.86 (s, 2H), 3.86 (s, 3H). ^13^C NMR (101 MHz, DMSO-*d*_6_) δ 169.13, 166.46, 165.20, 162.73, 159.99, 144.93, 130.00, 129.91, 126.93, 126.76, 122.78, 116.32, 116.10, 114.92, 114.32, 55.60. LC-MS: *m/z* 353 [M+H]^+^.

Compound **14** (ID: 1032287) *1-(4-ethylphenyl)-4-[3-(4-fluorophenyl)-1,2,4-oxadiazol-5-yl]-1H-1,2,3-triazol-5-amine:* Yield 89%. ^1^H NMR (400 MHz, DMSO-*d*_6_) δ 8.26 (t, *J* = 6.0 Hz, 2H), 7.54 (d, *J* = 8.4 Hz, 2H), 7.47 (d, *J* = 8.5 Hz, 2H), 7.41 (t, *J* = 8.4 Hz, 2H), 6.93 (s, 2H), 2.71 (q, *J* = 7.4 Hz, 2H), 1.23 (td, *J* = 7.6, 2.0 Hz, 3H). ^13^C NMR (101 MHz, DMSO-*d*_6_) δ 169.58, 166.95, 145.92, 145.19, 132.47, 130.38, 129.58, 128.97, 125.20, 123.22, 116.80, 116.57, 28.31, 15.27. LC-MS: *m/z* 351 [M+H]^+^.

Compound **15** (ID: 1032267) *1-(3-fluorophenyl)-4-[3-(4-fluorophenyl)-1,2,4-oxadiazol-5-yl]-1H-1,2,3-triazol-5-amine:* Yield 83%. ^1^H NMR (400 MHz, DMSO-*d*_6_) δ 8.31 – 8.17 (m, 2H), 7.74 – 7.64 (m, 1H), 7.58 (d, *J* = 9.3 Hz, 1H), 7.51 (d, *J* = 8.1 Hz, 1H), 7.48 – 7.33 (m, 3H), 7.07 (s, 2H). ^13^C NMR (101 MHz, DMSO-*d*_6_) δ 169.42, 166.94, 165.69, 163.83, 163.21, 161.39, 145.25, 136.03, 135.92, 132.11, 130.44, 123.17, 121.54, 117.10, 116.76, 116.54, 115.06, 113.05, 112.81. LC-MS: *m/z* 341 [M+H]^+^.

Compound **17** (ID: 1032290) *1-(3-chloro-4-methoxyphenyl)-4-[3-(4-fluorophenyl)-1,2,4-oxadiazol-5-yl]-1H-1,2,3-triazol-5-amine:* Yield 82%. ^1^H NMR (400 MHz, DMSO-*d*_6_) δ 8.30 – 8.18 (m, 2H), 7.74 (s, 1H), 7.59 (d, *J* = 8.7 Hz, 1H), 7.41 (t, *J* = 8.2 Hz, 3H), 6.86 (s, 2H), 3.98 (s, 3H). ^13^C NMR (101 MHz, DMSO-*d*_6_) δ 169.06, 166.46, 165.21, 162.73, 155.47, 145.13, 130.00, 129.91, 127.15, 127.11, 125.90, 122.76, 122.73, 121.59, 116.32, 116.10, 114.19, 113.26, 56.60. LC-MS: *m/z* 387 [M+H]^+^.

Compound **19** (ID: 1032273) *4-[3-(4-fluorophenyl)-1,2,4-oxadiazol-5-yl]-1-[3-(trifluoromethyl)phenyl]-1H-1,2,3-triazol-5-amine:* Yield 81%. ^1^H NMR (400 MHz, DMSO-*d*_6_) δ 8.25 (t, *J* = 6.9 Hz, 2H), 8.02 (s, 1H), 8.00 – 7.92 (m, 2H), 7.88 (t, *J* = 8.0 Hz, 1H), 7.46 – 7.33 (m, 2H), 7.09 (s, 2H). ^13^C NMR (101 MHz, DMSO-*d*_6_) δ 169.44, 166.96, 163.22, 145.51, 135.40, 131.64, 131.07, 130.43, 129.59, 126.83, 123.17, 122.46, 116.78, 116.55, 115.11. LC-MS: *m/z* 391 [M+H]^+^.

Compound **20** (ID: 1032285) *5-[3-(4-Fluoro-phenyl)-[1,2,4]oxadiazol-5-yl]-3-(3-methoxy-phenyl)-3H-[1,2,3]triazol-4-ylamine:* Yield 69%. ^1^H NMR (400 MHz, DMSO-*d*_6_) δ 8.30 – 8.17 (m, 2H), 7.54 (t, *J* = 8.3 Hz, 1H), 7.39 (t, *J* = 8.7 Hz, 2H), 7.23 – 7.09 (m, 3H), 6.97 (s, 2H), 3.84 (s, 3H). ^13^C NMR (101 MHz, DMSO-*d*_6_) δ 169.51, 166.93, 165.68, 163.21, 160.45, 145.12, 135.71, 131.15, 130.45, 130.37, 123.21, 123.18, 117.21, 117.17, 116.77, 116.55, 116.00, 115.03, 110.65, 55.97. LC-MS: *m/z* 353 [M+H]^+^.

Compound **21** (ID: 1032279) *1-(3,4-dimethoxyphenyl)-4-[3-(4-fluorophenyl)-1,2,4-oxadiazol-5-yl]-1H-1,2,3-triazol-5-amine:* Yield 78%. ^1^H NMR (400 MHz, DMSO-*d*_6_) δ 8.29 – 8.19 (m, 2H), 7.39 (t, *J* = 8.7 Hz, 2H), 7.22 – 7.11 (m, 3H), 6.86 (s, 2H). ^13^C NMR (101 MHz, DMSO-*d*_6_) δ 169.62, 166.93, 165.67, 163.20, 150.13, 149.67, 149.63, 145.37, 130.43, 130.35, 127.33, 127.31, 123.23, 118.04, 116.76, 116.57, 116.54, 114.74, 112.40, 112.38, 112.35, 109.66, 109.62, 109.59, 56.27, 56.20. LC-MS: *m/z* 383 [M+H]^+^.

Compound **22** (ID: 1032293**)** *1-(2,4-difluorophenyl)-4-[3-(4-fluorophenyl)-1,2,4-oxadiazol-5-yl]-1H-1,2,3-triazol-5-amine:* Yield 85%. ^1^H NMR (400 MHz, DMSO-*d*_6_) δ 8.26 (dd, *J* = 8.6, 5.4 Hz, 2H), 7.88 – 7.75 (m, 1H), 7.74 – 7.64 (m, 1H), 7.46 – 7.32 (m, 3H), 7.17 (s, 2H). ^13^C NMR (101 MHz, DMSO-*d*_6_) δ 169.45, 166.99, 165.70, 165.12, 163.23, 162.63, 159.16, 159.02, 156.63, 156.50, 146.60, 131.55, 131.52, 131.45, 130.47, 130.38, 123.26, 123.23, 118.99, 118.96, 118.87, 118.83, 116.75, 116.64, 116.53, 114.13, 113.40, 113.38, 113.17, 106.50, 106.27, 106.25, 106.24, 106.00. LC-MS: *m/z* 359 [M+H]^+^.

Compound **23** (ID: 1032266) *1-(2-fluorophenyl)-4-[3-(4-fluorophenyl)-1,2,4-oxadiazol-5-yl]-1H-1,2,3-triazol-5-amine:* Yield 78%. ^1^H NMR (400 MHz, DMSO-*d*_6_) δ 8.26 (dd, *J* = 8.6, 5.4 Hz, 2H), 7.70 (q, *J* = 7.6 Hz, 2H), 7.57 (t, *J* = 9.3 Hz, 1H), 7.46 (t, *J* = 7.7 Hz, 1H), 7.40 (t, *J* = 8.7 Hz, 2H), 7.15 (s, 2H). ^13^C NMR (101 MHz, DMSO-*d*_6_) δ 169.47, 166.98, 165.70, 163.22, 158.37, 155.87, 146.38, 133.22, 130.47, 130.39, 129.79, 126.04, 123

Compound **24** (ID: 1032281) *1-(3-chloro-2-methylphenyl)-4-[3-(4-fluorophenyl)-1,2,4-oxadiazol-5-yl]-1H-1,2,3-triazol-5-amine:* Yield 80%. ^1^H NMR (400 MHz, DMSO-*d*_6_) δ 8.26 (t, *J* = 6.0 Hz, 2H), 7.73 (t, *J* = 4.7 Hz, 1H), 7.54 – 7.44 (m, 2H), 7.39 (t, *J* = 8.7 Hz, 2H), 6.99 (s, 2H), 2.08 (s, 3H). ^13^C NMR (101 MHz, DMSO-*d*_6_) δ 169.54, 166.96, 165.69, 163.21, 146.08, 135.14, 134.69, 131.79, 130.45, 128.66, 127.68, 123.25, 116.74, 116.52, 114.28, 15.33. LC-MS: *m/z* 371 [M+H]^+^..25, 122.22, 122.10, 117.72, 117.54, 116.75, 116.53, 114.22. LC-MS: *m/z* 341 [M+H]^+^.

Compound **26** (ID: 1032276) *4-[3-(4-fluorophenyl)-1,2,4-oxadiazol-5-yl]-1-(2-methoxyphenyl)-1H-1,2,3-triazol-5-amine:* Yield 88%. ^1^H NMR (400 MHz, DMSO-*d*_6_) δ 8.32 – 8.19 (m, 2H), 7.60 (t, *J* = 8.0 Hz, 1H), 7.49 (d, *J* = 7.4 Hz, 1H), 7.40 (t, *J* = 8.6 Hz, 2H), 7.31 (d, *J* = 8.5 Hz, 1H), 7.15 (t, *J* = 7.7 Hz, 1H), 6.81 (s, 2H), 3.82 (s, 3H). ^13^C NMR (101 MHz, DMSO-*d*_6_) δ 169.69, 166.92, 163.20, 154.79, 146.22, 132.47, 130.44, 129.16, 123.33, 122.74, 121.24, 116.75, 116.53, 114.13, 113.50, 56.29. LC-MS: *m/z* 353 [M+H]^+^.

Compound **27** (ID: 1032282) *1-(5-chloro-2-methoxyphenyl)-4-[3-(4-fluorophenyl)-1,2,4-oxadiazol-5-yl]-1H-1,2,3-triazol-5-amine:* Yield 81%. ^1^H NMR (400 MHz, DMSO-*d*_6_) δ 8.27 (dd, *J* = 8.7, 5.6 Hz, 2H), 7.73 – 7.62 (m, 2H), 7.42 (t, *J* = 8.8 Hz, 2H), 7.38 – 7.32 (m, 1H), 6.94 (s, 2H), 3.83 (s, 3H). ^13^C NMR (101 MHz, DMSO-*d*_6_) δ 169.09, 166.45, 165.20, 162.72, 153.57, 145.83, 131.70, 130.01, 129.92, 128.57, 123.94, 123.13, 122.82, 122.79, 116.30, 116.08, 114.63, 113.49, 56.27. LC-MS: *m/z* 387 [M+H]^+^.

Compound **29** (ID: 1032278) *1-(2,5-dimethoxyphenyl)-4-[3-(4-fluorophenyl)-1,2,4-oxadiazol-5-yl]-1H-1,2,3-triazol-5-amine:* Yield 87%. ^1^H NMR (400 MHz, DMSO-*d*_6_) δ 8.31 – 8.20 (m, 2H), 7.40 (t, *J* = 8.4 Hz, 2H), 7.24 (dd, *J* = 9.2, 2.3 Hz, 1H), 7.17 (dd, *J* = 9.2, 2.8 Hz, 1H), 7.14 – 7.10 (m, 1H), 6.83 (s, 2H), 3.76 (s, 6H). ^13^C NMR (101 MHz, DMSO-*d*_6_) δ 169.69, 166.94, 165.68, 163.21, 153.58, 148.89, 146.21, 130.44, 123.33, 122.97, 117.79, 116.75, 116.53, 114.51, 114.11, 56.67, 56.30. LC-MS: *m/z* 383 [M+H]^+^.

Compound **31** (ID: 1032260) *1-(4-bromo-2-fluorophenyl)-4-[3-(4-chlorophenyl)-1,2,4-oxadiazol-5-yl]-1H-1,2,3-triazol-5-amine:* Yield 76%. ^1^H NMR (400 MHz, DMSO-*d*_6_) δ 8.23 (d, *J* = 8.2 Hz, 2H), 7.99 (d, *J* = 9.5 Hz, 1H), 7.76 – 7.68 (m, 2H), 7.64 (d, *J* = 8.4 Hz, 2H), 7.22 (s, 2H). ^13^C NMR (101 MHz, DMSO-*d*_6_) δ 168.97, 166.50, 157.94, 155.38, 146.01, 136.27, 130.86, 129.20, 129.18, 128.85, 128.81, 125.04, 124.49, 124.40, 121.26, 121.14, 120.82, 120.60, 113.62, 40.36. LC-MS: *m/z* 435 [M+H]^+^.

Compound **32** (ID: 1032298) *4-[3-(4-bromophenyl)-1,2,4-oxadiazol-5-yl]-1-(4-fluorophenyl)-1H-1,2,3-triazol-5-amine:* Yield 83%. ^1^H NMR (400 MHz, DMSO-*d*_6_) δ 8.16 (d, *J* = 8.0 Hz, 2H), 7.80 (d, *J* = 8.0 Hz, 2H), 7.75 – 7.65 (m, 2H), 7.56 – 7.46 (m, 2H), 7.00 (s, 2H). ^13^C NMR (101 MHz, DMSO-*d*_6_) δ 169.14, 166.60, 163.56, 161.11, 145.03, 132.14, 130.57, 129.37, 127.79, 127.70, 125.38, 125.17, 116.88, 116.65, 114.36. LC-MS: *m/z* 401 [M+H]^+^.

Compound **33** (ID: 1032297) *4-[3-(4-bromophenyl)-1,2,4-oxadiazol-5-yl]-1-(3-fluorophenyl)-1H-1,2,3-triazol-5-amine:* Yield 87%. ^1^H NMR (400 MHz, DMSO-*d*_6_) δ 8.13 (d, *J* = 8.1 Hz, 2H), 7.76 (d, *J* = 8.1 Hz, 2H), 7.69 (q, *J* = 7.3, 6.8 Hz, 1H), 7.58 (d, *J* = 8.9 Hz, 1H), 7.51 (d, *J* = 8.0 Hz, 1H), 7.45 (t, *J* = 8.3 Hz, 1H), 7.08 (s, 2H). ^13^C NMR (101 MHz, DMSO-*d*_6_) δ 169.55, 167.09, 163.84, 161.39, 145.29, 136.03, 135.92, 132.60, 132.10, 132.04, 129.82, 125.84, 125.63, 121.55, 117.12, 116.90, 115.04, 113.06, 113.05, 112.82. LC-MS: *m/z* 401 [M+H]^+^.

Compound **34** (ID: 1032296) *4-[3-(4-bromophenyl)-1,2,4-oxadiazol-5-yl]-1-(2-fluorophenyl)-1H-1,2,3-triazol-5-amine:* Yield 80%. ^1^H NMR (400 MHz, DMSO-*d*_6_) δ 8.16 (d, *J* = 8.6 Hz, 2H), 7.79 (d, *J* = 8.5 Hz, 2H), 7.75 – 7.68 (m, 2H), 7.62 – 7.55 (m, 1H), 7.48 (t, *J* = 7.7 Hz, 1H). ^13^C NMR (101 MHz, DMSO-*d*_6_) δ 169.06, 166.62, 157.89, 155.38, 145.92, 132.79, 132.71, 132.13, 129.39, 129.35, 125.59, 125.56, 125.41, 125.17, 121.71, 121.58, 117.27, 117.08, 113.66. LC-MS: *m/z* 401 [M+H]^+^.

Compound **37** (ID: 1032262) *1-(5-chloro-2-methoxyphenyl)-4-[3-(4-methoxyphenyl)-1,2,4-oxadiazol-5-yl]-1H-1,2,3-triazol-5-amine:* Yield 78%. ^1^H NMR (400 MHz, DMSO-*d*_6_) δ 8.14 (d, *J* = 8.8 Hz, 2H), 7.72 – 7.62 (m, 2H), 7.35 (d, *J* = 9.7 Hz, 1H), 7.12 (d, *J* = 8.6 Hz, 2H), 6.89 (s, 2H), 3.85 (s, 3H), 3.83 (s, 3H). ^13^C NMR (101 MHz, DMSO-*d*_6_) δ 168.79, 166.94, 161.69, 153.57, 145.74, 131.66, 129.08, 128.54, 123.97, 123.21, 118.52, 114.65, 114.44, 113.67, 56.30, 55.38. LC-MS: *m/z* 399 [M+H]^+^.

Compound **39** (ID: 1032312) *4-[3-(4-ethoxyphenyl)-1,2,4-oxadiazol-5-yl]-1-(2-fluorophenyl)-1H-1,2,3-triazol-5-amine:* Yield 78%.^1^H NMR (400 MHz, DMSO-*d*_6_) δ 8.12 (d, *J* = 8.2 Hz, 2H), 7.70 (q, *J* = 7.2 Hz, 2H), 7.57 (t, *J* = 9.4 Hz, 1H), 7.46 (t, *J* = 7.7 Hz, 1H), 7.15 – 7.04 (m, 4H), 4.10 (q, *J* = 7.0 Hz, 2H), 1.34 (t, *J* = 6.9 Hz, 3H).^13^C NMR (101 MHz, DMSO-*d*_6_) δ 169.09, 167.45, 161.47, 158.35, 155.85, 146.26, 133.24, 133.16, 129.85, 129.76, 129.63, 129.59, 129.55, 126.06, 126.04, 126.03, 126.01, 122.22, 122.09, 117.73, 117.54, 115.27, 114.30, 63.82. LC-MS: *m/z* 367 [M+H]^+^.

Compound **40** (ID: 1032315) *1-(2-chlorophenyl)-4-[3-(4-ethoxyphenyl)-1,2,4-oxadiazol-5-yl]-1H-1,2,3-triazol-5-amine:* Yield 85%. ^1^H NMR (400 MHz, DMSO-*d*_6_) δ 8.11 (d, *J* = 8.4 Hz, 2H), 7.79 (d, *J* = 8.0 Hz, 1H), 7.75 – 7.65 (m, 2H), 7.60 (t, *J* = 7.7 Hz, 1H), 7.12 – 6.99 (m, 4H), 4.09 (q, *J* = 6.9 Hz, 2H), 1.34 (t, *J* = 6.9 Hz, 3H). ^13^C NMR (101 MHz, DMSO-*d*_6_) δ 169.17, 167.47, 161.47, 146.23, 132.96, 131.97, 131.91, 131.02, 130.75, 129.59, 129.13, 118.79, 115.27, 114.14, 63.82, 14.98. LC-MS: *m/z* 383 [M+H]^+^.

Compound **41** (ID: 1032333) *1-(3-chloro-4-fluorophenyl)-4-[3-(4-ethoxyphenyl)-1,2,4-oxadiazol-5-yl]-1H-1,2,3-triazol-5-amine:* Yield 78%. ^1^H NMR (400 MHz, DMSO-*d*_6_) δ 8.10 (d, *J* = 7.8 Hz, 2H), 7.97 (d, *J* = 5.3 Hz, 1H), 7.69 (d, *J* = 6.7 Hz, 2H), 7.07 (d, *J* = 8.4 Hz, 2H), 7.01 (s, 2H), 4.10 (q, *J* = 6.9 Hz, 2H), 1.34 (t, *J* = 7.0 Hz, 3H). ^13^C NMR (101 MHz, DMSO-*d*_6_) δ 169.14, 167.48, 161.50, 159.43, 156.96, 145.54, 131.70, 131.67, 129.56, 128.54, 128.52, 127.15, 127.08, 121.17, 120.98, 118.75, 118.56, 118.33, 115.33, 114.98, 63.84, 14.97. LC-MS: *m/z* 401 [M+H]^+^.

Spectra for 11 compounds: **6** (ID: 1032350), **8** (ID: 1032340), **10** (ID: 1032271), **16** (ID: 1032270), **18** (ID: 1032264), **25** (ID: 1032283), **28** (ID: 1032284), **30** (ID: 1032286), **35** (ID: 1032301), **36** (ID: 1032306), **38** (ID: 1032369) are not presented due to low amount of synthesized compound to obtain ^13^C NMR. ^1^H NMR and LC-MS were performed for these compounds (data not shown).

**Supplementary Notes**

**Potential of Mean Force (PMF) profile for LeuRS**

# This file was created Fri Aug 7 11:33:18 2020

# by the following command:

# g_wham -it tpr-files.dat -if pullf-files.dat -o -hist -unit kCal

#

# g_wham is part of G R O M A C S:

#

# Great Red Owns Many ACres of Sand

#

@ title "Umbrella potential"

@ xaxis label "z"

@ yaxis label "E (kcal mol\S-1\N)"

@TYPE xy

9.000979e-01 nan

9.166380e-01 nan

9.331780e-01 nan

9.497180e-01 nan

9.662580e-01 nan

9.827980e-01 nan

9.993380e-01 nan

1.015878e+00 nan

1.032418e+00 nan

1.048958e+00 nan

1.065498e+00 nan

1.082038e+00 nan

1.098578e+00 nan

1.115118e+00 nan

1.131658e+00 nan

1.148198e+00 nan

1.164738e+00 nan

1.181278e+00 nan

1.197818e+00 nan

1.214358e+00 nan

1.230898e+00 nan

1.247438e+00 nan

1.263978e+00 nan

1.280518e+00 nan

1.297058e+00 nan

1.313598e+00 nan

1.330138e+00 nan

1.346678e+00 nan

1.363218e+00 nan

1.379758e+00 nan

1.396298e+00 nan

1.412838e+00 nan

1.429378e+00 nan

1.445918e+00 nan

1.462458e+00 nan

1.478998e+00 nan

1.495538e+00 nan

1.512078e+00 nan

1.528618e+00 nan

1.545158e+00 nan

1.561698e+00 nan

1.578238e+00 nan

1.594778e+00 nan

1.611318e+00 nan

1.627858e+00 nan

1.644399e+00 nan

1.660939e+00 nan

1.677479e+00 nan

1.694019e+00 nan

1.710559e+00 nan

1.727099e+00 nan

1.743639e+00 nan

1.760179e+00 nan

1.776719e+00 nan

1.793259e+00 nan

1.809799e+00 nan

1.826339e+00 nan

1.842879e+00 nan

1.859419e+00 nan

1.875959e+00 nan

1.892499e+00 nan

1.909039e+00 nan

1.925579e+00 nan

1.942119e+00 nan

1.958659e+00 nan

1.975199e+00 nan

1.991739e+00 nan

2.008279e+00 nan

2.024819e+00 nan

2.041359e+00 nan

2.057899e+00 nan

2.074439e+00 nan

2.090979e+00 nan

2.107519e+00 nan

2.124059e+00 nan

2.140599e+00 nan

2.157139e+00 nan

2.173679e+00 nan

2.190219e+00 nan

2.206759e+00 nan

2.223299e+00 nan

2.239839e+00 nan

2.256379e+00 nan

2.272919e+00 nan

2.289459e+00 nan

2.305999e+00 nan

2.322539e+00 nan

2.339079e+00 nan

2.355619e+00 nan

2.372159e+00 nan

2.388699e+00 nan

2.405239e+00 nan

2.421779e+00 nan

2.438319e+00 nan

2.454859e+00 nan

2.471399e+00 nan

2.487939e+00 nan

2.504479e+00 nan

2.521019e+00 nan

2.537559e+00 nan

2.554099e+00 nan

2.570639e+00 nan

2.587179e+00 nan

2.603719e+00 nan

2.620259e+00 nan

2.636799e+00 nan

2.653339e+00 nan

2.669879e+00 nan

2.686419e+00 nan

2.702959e+00 nan

2.719499e+00 nan

2.736039e+00 nan

2.752579e+00 nan

2.769119e+00 nan

2.785659e+00 nan

2.802199e+00 nan

2.818739e+00 nan

2.835279e+00 nan

2.851819e+00 nan

2.868359e+00 nan

2.884899e+00 nan

2.901439e+00 nan

2.917979e+00 nan

2.934519e+00 nan

2.951059e+00 nan

2.967599e+00 nan

2.984140e+00 nan

3.000680e+00 nan

3.017220e+00 nan

3.033760e+00 nan

3.050300e+00 nan

3.066840e+00 nan

3.083380e+00 nan

3.099920e+00 nan

3.116460e+00 nan

3.133000e+00 nan

3.149540e+00 nan

3.166080e+00 nan

3.182620e+00 nan

3.199160e+00 nan

3.215700e+00 nan

3.232240e+00 nan

3.248780e+00 nan

3.265320e+00 nan

3.281860e+00 nan

3.298400e+00 nan

3.314940e+00 nan

3.331480e+00 nan

3.348020e+00 nan

3.364560e+00 nan

3.381100e+00 nan

3.397640e+00 nan

3.414180e+00 nan

3.430720e+00 nan

3.447260e+00 nan

3.463800e+00 nan

3.480340e+00 nan

3.496880e+00 nan

3.513420e+00 nan

3.529960e+00 nan

3.546500e+00 nan

3.563040e+00 nan

3.579580e+00 nan

3.596120e+00 nan

3.612660e+00 nan

3.629200e+00 nan

3.645740e+00 nan

3.662280e+00 nan

3.678820e+00 nan

3.695360e+00 nan

3.711900e+00 nan

3.728440e+00 nan

3.744980e+00 nan

3.761520e+00 nan

3.778060e+00 nan

3.794600e+00 nan

3.811140e+00 nan

3.827680e+00 nan

3.844220e+00 nan

3.860760e+00 nan

3.877300e+00 nan

3.893840e+00 nan

3.910380e+00 nan

3.926920e+00 nan

3.943460e+00 nan

3.960000e+00 nan

3.976540e+00 nan

3.993080e+00 nan

4.009620e+00 nan

4.026160e+00 nan

4.042700e+00 nan

4.059240e+00 nan

4.075780e+00 nan

4.092320e+00 nan

4.108860e+00 nan

4.125400e+00 nan

4.141940e+00 nan

4.158480e+00 nan

4.175020e+00 nan

4.191560e+00 nan

**Supplementary Notes**

**Potential of Mean Force (PMF) for MetRS**

# This file was created Wed Jul 17 11:54:51 2019

# by the following command:

# g_wham -it tpr-files.dat -if pullf-files.dat -o -hist -unit kCal

#

# g_wham is part of G R O M A C S:

#

# Great Red Owns Many ACres of Sand

#

@ title "Umbrella potential"

@ xaxis label "z"

@ yaxis label "E (kcal mol\S-1\N)"

@TYPE xy

1.321443e+00 5.086914e-01

1.331687e+00 4.911831e-01

1.341931e+00 5.500655e-01

1.352176e+00 4.579232e-01

1.362420e+00 4.608989e-01

1.372664e+00 4.475507e-01

1.382909e+00 5.008821e-01

1.393153e+00 7.136051e-01

1.403398e+00 8.017621e-01

1.413642e+00 9.955793e-01

1.659506e+00 9.692364e-01

1.669751e+00 7.498109e-01

1.679995e+00 5.494765e-01

1.690240e+00 5.337059e-01

1.700484e+00 6.064496e-01

1.710728e+00 8.554330e-01

1.720973e+00 7.396902e-01

1.731217e+00 4.936271e-01

1.741461e+00 9.250683e-01

1.751706e+00 6.444331e-01

1.761950e+00 6.561566e-01

1.772194e+00 6.381717e-01

1.782439e+00 6.241295e-01

1.792683e+00 8.277486e-01

1.802927e+00 9.158837e-01

1.813172e+00 6.917512e-01

1.823416e+00 1.012513e+00

1.833660e+00 8.234106e-01

1.843905e+00 9.526759e-01

1.854149e+00 1.114465e+00

1.864394e+00 1.109034e+00

1.874638e+00 9.826444e-01

1.884882e+00 1.099905e+00

1.895127e+00 1.080167e+00

1.905371e+00 9.372347e-01

1.915615e+00 8.958238e-01

1.925860e+00 1.025465e+00

1.936104e+00 1.076885e+00

1.946348e+00 1.229974e+00

1.956593e+00 1.341756e+00

1.966837e+00 1.430002e+00

1.977081e+00 1.536816e+00

1.987326e+00 1.586446e+00

1.997570e+00 1.513243e+00

2.007815e+00 1.614628e+00

2.018059e+00 1.784000e+00

2.028303e+00 1.737630e+00

2.038548e+00 1.824214e+00

2.048792e+00 1.821987e+00

2.059036e+00 1.873540e+00

2.069281e+00 1.843216e+00

2.079525e+00 1.830087e+00

2.089769e+00 1.883871e+00

2.100014e+00 2.115274e+00

2.110258e+00 2.065801e+00

2.120502e+00 2.173784e+00

2.130747e+00 2.401764e+00

2.140991e+00 2.121702e+00

2.151236e+00 2.103469e+00

2.161480e+00 2.121092e+00

2.171724e+00 2.169092e+00

2.181969e+00 2.137364e+00

2.192213e+00 2.015682e+00

2.202457e+00 2.227744e+00

2.212702e+00 2.098983e+00

2.222946e+00 1.935235e+00

2.233190e+00 1.942150e+00

2.243435e+00 2.071066e+00

2.253679e+00 1.976978e+00

2.263923e+00 1.871871e+00

2.274168e+00 2.015208e+00

2.284412e+00 2.121490e+00

2.294657e+00 2.280630e+00

2.304901e+00 2.395532e+00

2.315145e+00 2.342401e+00

2.325390e+00 2.348022e+00

2.335634e+00 2.873730e+00

2.345878e+00 2.447643e+00
